# Sine oculis homeobox homolog 1 plays a critical role in pulmonary fibrosis

**DOI:** 10.1172/jci.insight.142984

**Published:** 2022-05-23

**Authors:** Cory Wilson, Tinne C.J. Mertens, Pooja Shivshankar, Weizen Bi, Scott D. Collum, Nancy Wareing, Junsuk Ko, Tingting Weng, Ram P. Naikawadi, Paul J. Wolters, Pascal Maire, Soma S.K. Jyothula, Rajarajan A. Thandavarayan, Dewei Ren, Nathan D. Elrod, Eric J. Wagner, Howard J. Huang, Burton F. Dickey, Heide L. Ford, Harry Karmouty-Quintana

**Affiliations:** 1 Department of Biochemistry and Molecular Biology, McGovern Medical School, The University of Texas Health Science Center at Houston (UTHealth Houston), Houston, Texas, USA.; 2 Pulmonary, Critical Care, Allergy and Sleep Medicine, UCSF, San Francisco, California, USA.; 3 Université de Paris Cité, Institut Cochin, INSERM, CNRS, Paris, France.; 4 Divisions of Critical Care, Pulmonary and Sleep Medicine, Department of Internal Medicine, McGovern Medical School, UTHealth, Houston, Texas, USA.; 5 Methodist J.C. Walter Jr. Transplant Center, Houston Methodist Hospital, Houston, Texas, USA.; 6 Department of Biochemistry and Molecular Biology, University of Texas Medical Branch at Galveston, Galveston, Texas, USA.; 7 Department of Biochemistry and Biophysics, Center for RNA Biology, Wilmot Cancer Institute, University of Rochester School of Medicine and Dentistry, KMRB G.9629, Rochester, New York, USA.; 8 Department of Pulmonary Medicine, Division of Internal Medicine, The University of Texas M.D. Anderson Cancer Center, Houston, Texas, USA.; 9 Department of Pharmacology, University of Colorado, Anschutz Medical Campus, Aurora, Colorado, USA.

**Keywords:** Pulmonology, Fibrosis

## Abstract

Idiopathic pulmonary fibrosis (IPF) is a fatal disease with limited treatment options. The role of the developmental transcription factor Sine oculis homeobox homolog 1 (SIX1) in the pathophysiology of lung fibrosis is not known. IPF lung tissue samples and IPF-derived alveolar type II cells (AT2) showed a significant increase in *SIX1* mRNA and protein levels, and the SIX1 transcriptional coactivators *EYA1* and *EYA2* were elevated. *Six1* was also upregulated in bleomycin-treated (BLM-treated) mice and in a model of spontaneous lung fibrosis driven by deletion of Telomeric Repeat Binding Factor 1 (*Trf1*) in AT2 cells. Conditional deletion of *Six1* in AT2 cells prevented or halted BLM-induced lung fibrosis, as measured by a significant reduction in histological burden of fibrosis, reduced fibrotic mediator expression, and improved lung function. These effects were associated with increased macrophage migration inhibitory factor (MIF) in lung epithelial cells in vivo following *SIX1* overexpression in BLM-induced fibrosis. A MIF promoter–driven luciferase assay demonstrated direct binding of *Six1* to the 5′-TCAGG-3′ consensus sequence of the MIF promoter, identifying a likely mechanism of SIX1-driven MIF expression in the pathogenesis of lung fibrosis and providing a potentially novel pathway for targeting in IPF therapy.

## Graphical abstract

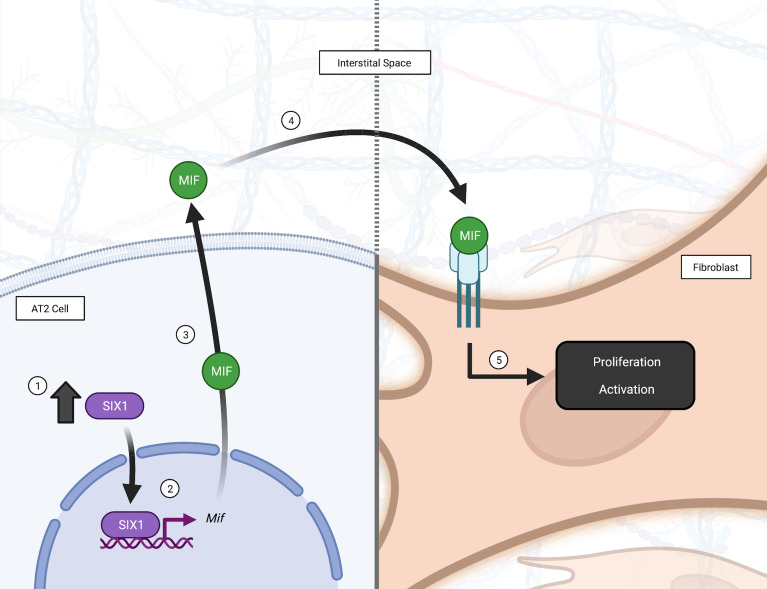

## Introduction

Idiopathic pulmonary fibrosis (IPF) is the most common type of interstitial lung disease, with a median survival of 2–4 years from the time of diagnosis (1). It is estimated that the prevalence of IPF in the United States is 10–60 cases per 100,000 people, with limited pharmacological therapies available (2, 3). IPF is a chronic, progressive disease characterized by alveolar injury, increased extracellular matrix (ECM) deposition and resultant alveolar destruction. Macroscopically, this leads to poor lung compliance, impaired transalveolocapillary membrane gas exchange and, ultimately, end-stage respiratory failure, necessitating lung transplantation (2, 4, 5). Several nongenetic risk factors, such as older age, and smoking, increase the risk of developing IPF (4, 6). More recently, several genetic risk factors for IPF have also been discovered, including a single-nucleotide polymorphism (rs35705950) in the promoter region of *MUC5B* (7, 8), which codes for an essential protein for airway clearance and innate immune response, along with genes associated with telomere maintenance, such as telomerase RNA component (*TERC*) and telomerase reverse transcriptase (*TERT*; refs. 1, 9).

The current paradigm for the pathogenesis of IPF postulates that chronic injury to the alveolar epithelium, specifically alveolar type II (AT2) cells, leads to aberrant tissue repair and fibrosis. Several studies (10–12) have shown that the reactivation of developmentally expressed genes in AT2 cells are associated with the abnormal epithelial phenotype seen in IPF. In this study, we aim to characterize one such developmental transcription factor, sine oculis homeobox homolog 1 (*SIX1*), which plays a role in lung development.

SIX1 is critical for the development of the alveolar epithelium and is present through the saccular stage of lung development in mice (13). The *SIX* family is composed of genes *SIX1–6*, all of which are expressed in both mice and humans and are characterized by a conserved protein interacting *SIX* domain (SD; 110–115 amino acids) and a DNA-binding homeobox domain (HD; 60 amino acids; ref. 14). SIX1 functions as a DNA-binding transcription factor in a complex with transcriptional coactivators and corepressors, most notably the Eyes absent (EYA) and Dachshund (DACH) families, respectively (14). Specifically, EYA1 and EYA2 are known transcriptional coactivators of SIX1 and have been well studied in breast cancer (15). Pathological expression of SIX1 has also been shown to promote the invasion of non-small cell lung cancer (NSCLC; refs. 16, 17). Importantly in the context of IPF, increased *SIX1* levels have been reported in lung tissue from patients with IPF (18, 19), as well as in an AT2-specific single-cell RNA-Seq (scRNA-Seq) data set that shows increased AT2 (CD326^+^HTII-280^+^) cell-specific expression of *SIX1* in patients with IPF (20). However, these studies did not demonstrate whether *SIX1* is expressed at the protein level or whether it contributes to lung fibrosis. Thus, we hypothesize that SIX1 expression is elevated in IPF and that it is a molecular driver of lung fibrosis that can be targeted therapeutically.

## Results

**Figure 1:**
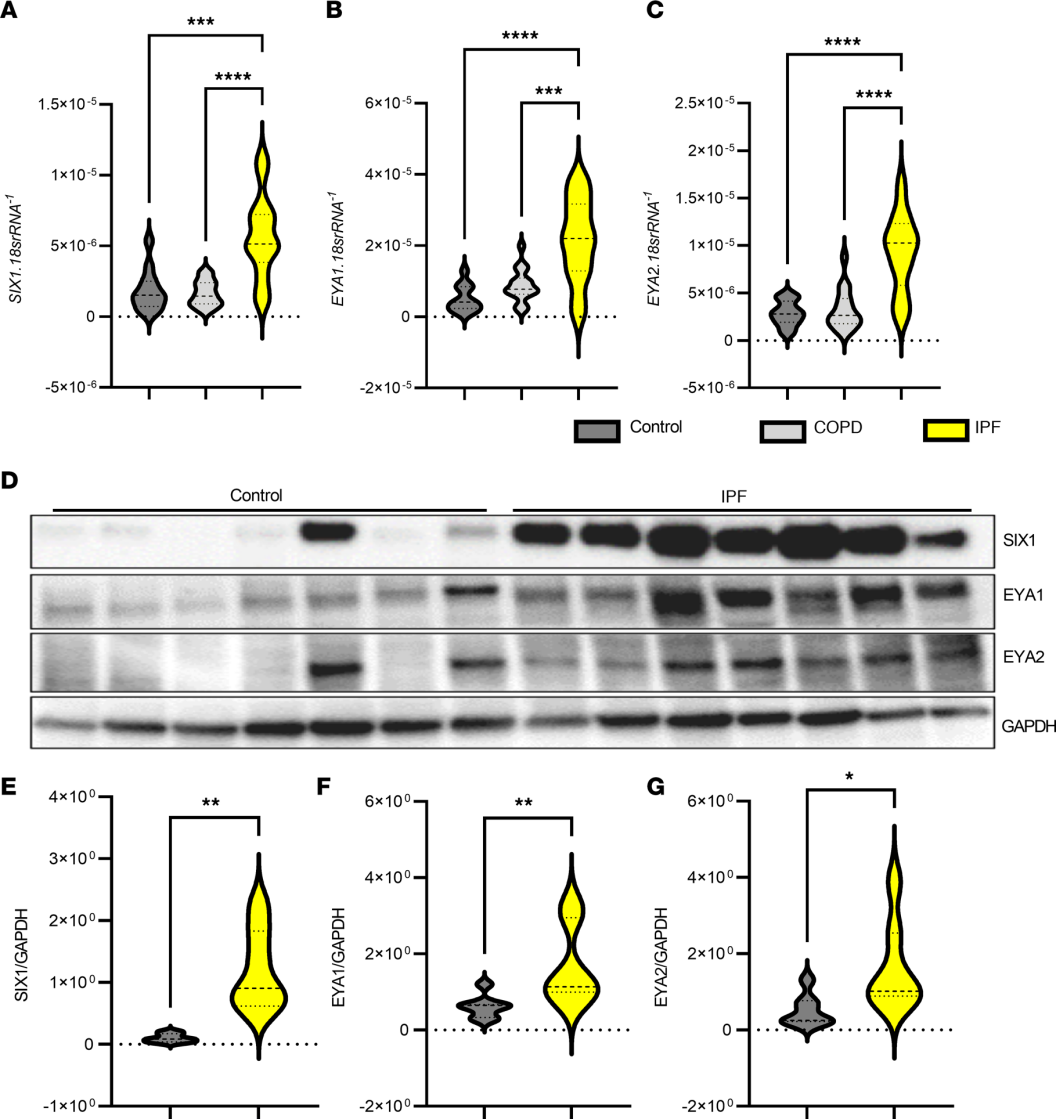
Increased SIX1, EYA1 and EYA2 in IPF. (**A**–**C**) qPCR for *SIX1* (**A**), *EYA1* (**B**), and *EYA2* (**C**) mRNA expression in IPF (*n =* 21) compared with COPD (*n =* 18) and control lungs (*n =* 12). Values represented here are ratios of the respective mRNA levels to that of 18srRNA control in these samples. (**D**) Western blot showing protein expression of SIX1, EYA1, and EYA2 in IPF (*n =* 7) versus control lungs (*n =* 7). (**E**–**G**) Densitometric analyses of the Western blots are represented as ratios of SIX1 (**E**), EYA1 (**F**), and EYA2 (**G**) with the loading control GAPDH. *P ≤ 0.05; **P ≤ 0.01; ***P ≤ 0.001, ****P ≤ 0.0001 refer to Kruskal-Wallis test with the 2-stage linear step-up procedure of Benjamini, Krieger, and Yekutieli post hoc test for transcript data and Mann-Whitney *U* test for densitometries.

SIX1 and its transcriptional coactivators EYA1 and EYA2 are elevated in IPF. Increased expression of *SIX1* has been reported in lung tissue and isolated AT2 cells by large discovery-based studies from independent groups (19, 20). Thus, to validate increased expression of SIX1 in IPF, we quantified the expression of *SIX1*, *EYA1*, and *EYA2* mRNA by quantitative PCR (qPCR) in IPF (*n =* 24), COPD (*n =* 18), and controls (*n =* 12). These experiments revealed a significant increase in *SIX1* (3-fold), *EYA1* (4-fold), and *EYA2* (3-fold) transcript levels in IPF but not in COPD or controls (Figure 1, A–C). The increased transcript levels for *SIX1*, *EYA1*, and *EYA2* were consistent with significantly elevated protein levels of SIX1, EYA1, and EYA2 in IPF (*n =* 7) as compared with the controls (*n =* 7) (Figure 1, D–G). Intriguingly, only 1 control subject presented with increased *SIX1* and *EYA2* signals, and the clinical history of this patient was remarkable due to previous methamphetamine abuse.

### Increased SIX1 levels are localized to AT2 cells.

**Figure 2:**
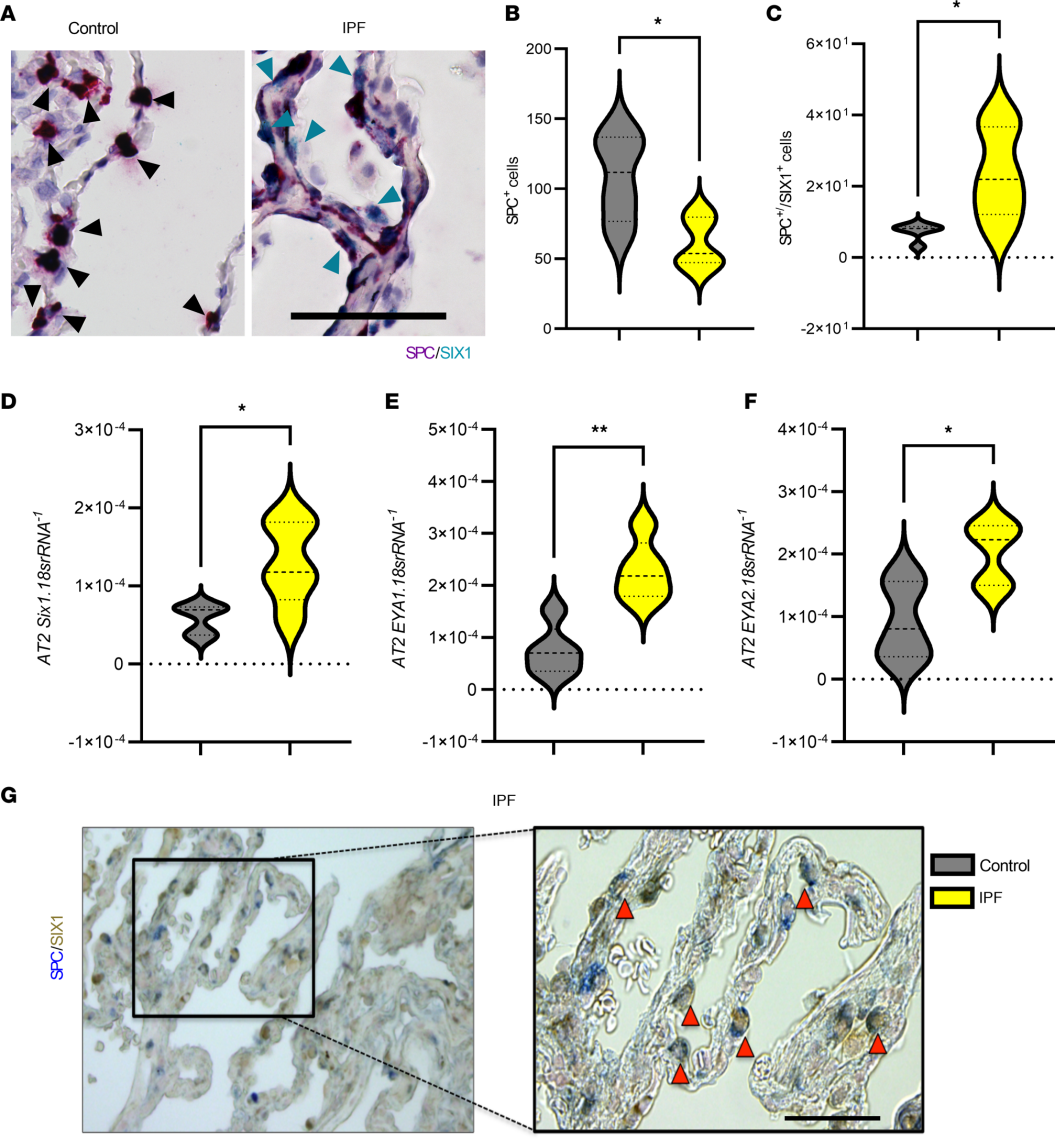
SIX1 signals are localized in lung AT2 cells in IPF. (**A**) Representative RNA in situ hybridization (RNAScope) for *SPC* (red) and *SIX1* (cyan) probes. Cyan arrowheads point at dual staining. Black arrowheads denote SPC-expressing cells. All samples are counterstained with hematoxylin. Scale bar: 50 μm. (**B** and **C**) Quantification of *SPC*^+^ cells (**B**) and dual-positive *SPC*^+^/*SIX1*^+^ cells (**C**) in control and IPF groups (*n =* 5 per group). In total, 7–10 field of views (FOVs) were selected from each tissue samples, and values represented are mean number of *SPC*^+^ cells per FOV. *SIX1*^+^ cells are represented as mean numbers per 100 SPC^+^ cells. (**D**–**F**) Expression levels of *SIX1* (**D**), *EYA1* (**E**), and *EYA2* (**F**), from isolated AT2 cells from control (*n =* 5) or IPF (*n =* 5) lungs. *P ≤ 0.05 and **P ≤ 0.01 refer to 2-tailed, unpaired Student’s *t* test with Welch’s correction. (**G**) Dual IHC staining of IPF lung tissue sections with expanded area showing type II alveolar epithelial cells (denoted with red arrowheads) with colocalization of surfactant protein C (blue) and SIX1 (red/brown). Scale bar: 50 μm.

In order to determine the cellular specificity of increased SIX1 expression in IPF, we performed RNA in situ hybridization to assess the localization of *SIX1* and AT2-specific surfactant protein C^+^ (SPC^+^) cell number (Figure 2, A–C). Our data highlight a reduction by half of SPC-expressing cells (Figure 2B) and a significant 3-fold increase of dual expression of SPC/SIX1 in IPF tissues compared with controls (Figure 2C). We further confirmed that isolated AT2 cells from patients with IPF exhibited significantly upregulated *SIX1* transcripts, along with expression of both *EYA1* and *EYA2* as compared with controls (Figure 2, D–F). IHC staining of IPF lung tissues for SIX1 protein (brown color) and SPC (blue color) showed AT2-specific colocalization of SIX1 with SPC (Figure 2G; red arrows). These results demonstrate that, in IPF, *SIX1*, *EYA1*, and *EYA2* expression levels are upregulated in AT2 cells and that SIX1 protein is increased in AT2 cells.

### SIX1 is upregulated in 2 distinct mouse models of pulmonary fibrosis.

**Figure 3:**
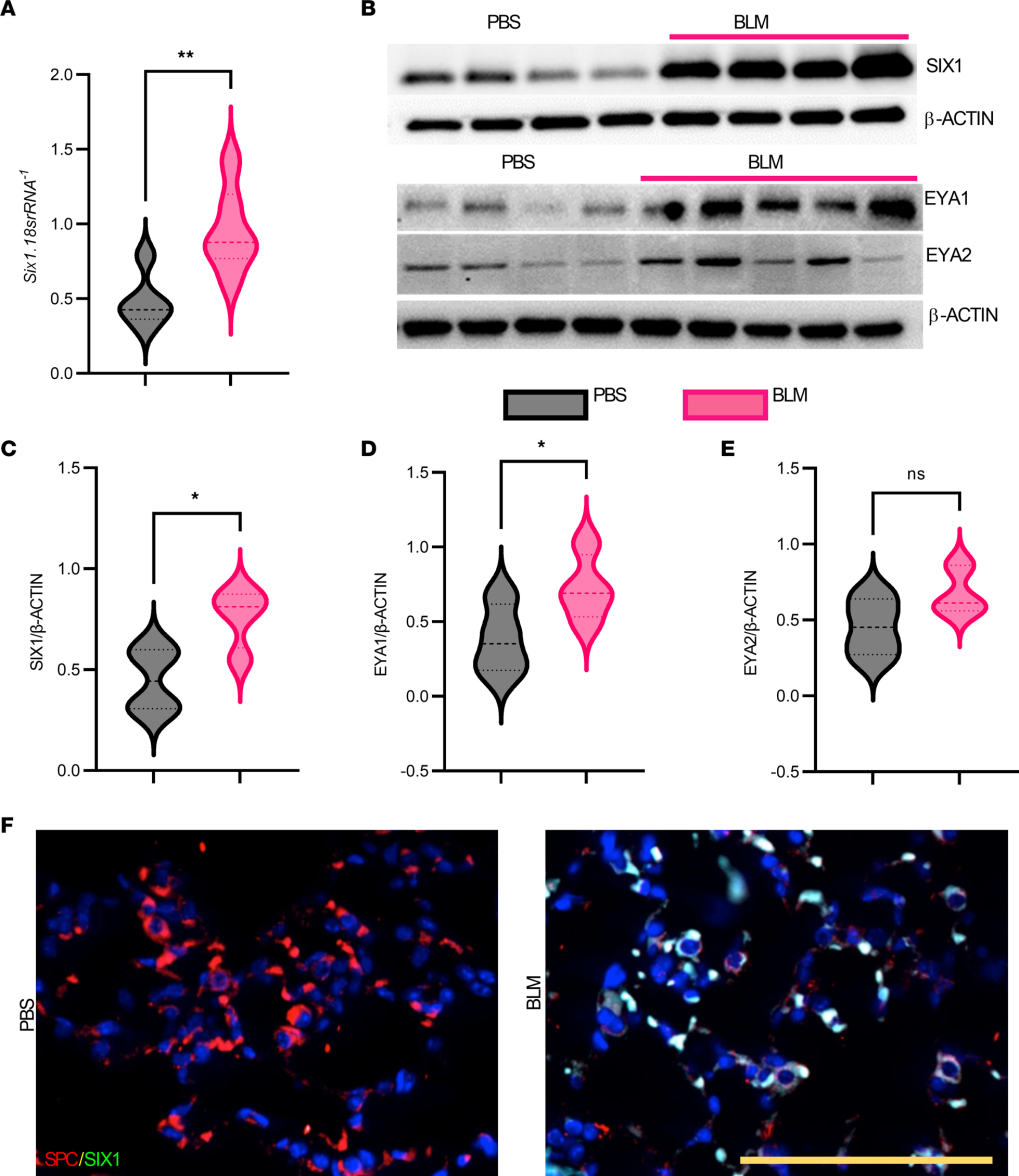
SIX1 and its cofactors are elevated in the BLM model of lung fibrosis. (**A** and **B**) *Six1* mRNA levels (*n =* 5) (**A**) and Western blots for SIX1, EYA1, and EYA2 (**B**) from C57BL/6 mice treated with 8 injections of bleomycin (BLM, 0.035 U/kg) or vehicle (PBS). (**C**–**E**) Densitometric quantitation for SIX1 (**C**), EYA1 (**D**), EYA2 (**E**) protein levels are shown as ratios of the respective proteins to that of actin, which served as a loading control. *P ≤ 0.05 and **P ≤ 0.01 refer to 2-tailed, unpaired Student’s *t* test with Welch’s correction. (**F**) Representative immunofluorescence images of SIX1 (cyan signal) expression and the colocalization with SPC^+^ (red signal) cells in lung tissue from PBS- (*n =* 5) and BLM-treated (*n =* 5) C57BL/6 mice. Scale bar: 50 μm.

The bleomycin (BLM) mouse model is a widely utilized system for the study of fibrotic lung disease (21). We used a chronic, low-dose i.p. model of BLM injury (22, 23); this has the advantage that fibrotic deposition is observed with subpleural distribution, which more closely resembles human disease with fibrotic scarring that is not typically reversible. Using this model, we observed a significant 2-fold increase in *Six1* transcript and protein levels (Figure 3, A–C). In line with this, we report increased levels of SIX1 cofactors: *Eya1* (1.5-fold) but not *Eya2* (Figure 3, B, D, and E). Lung immunofluorescence revealed increased SIX1 expression colocalized with SPC^+^ cells of BLM-treated mice but not in PBS controls (Figure 3F). Taken together, our data demonstrate increased expression of SIX1 in BLM-treated mice that is present in SPC-expressing cells.

**Figure 4:**
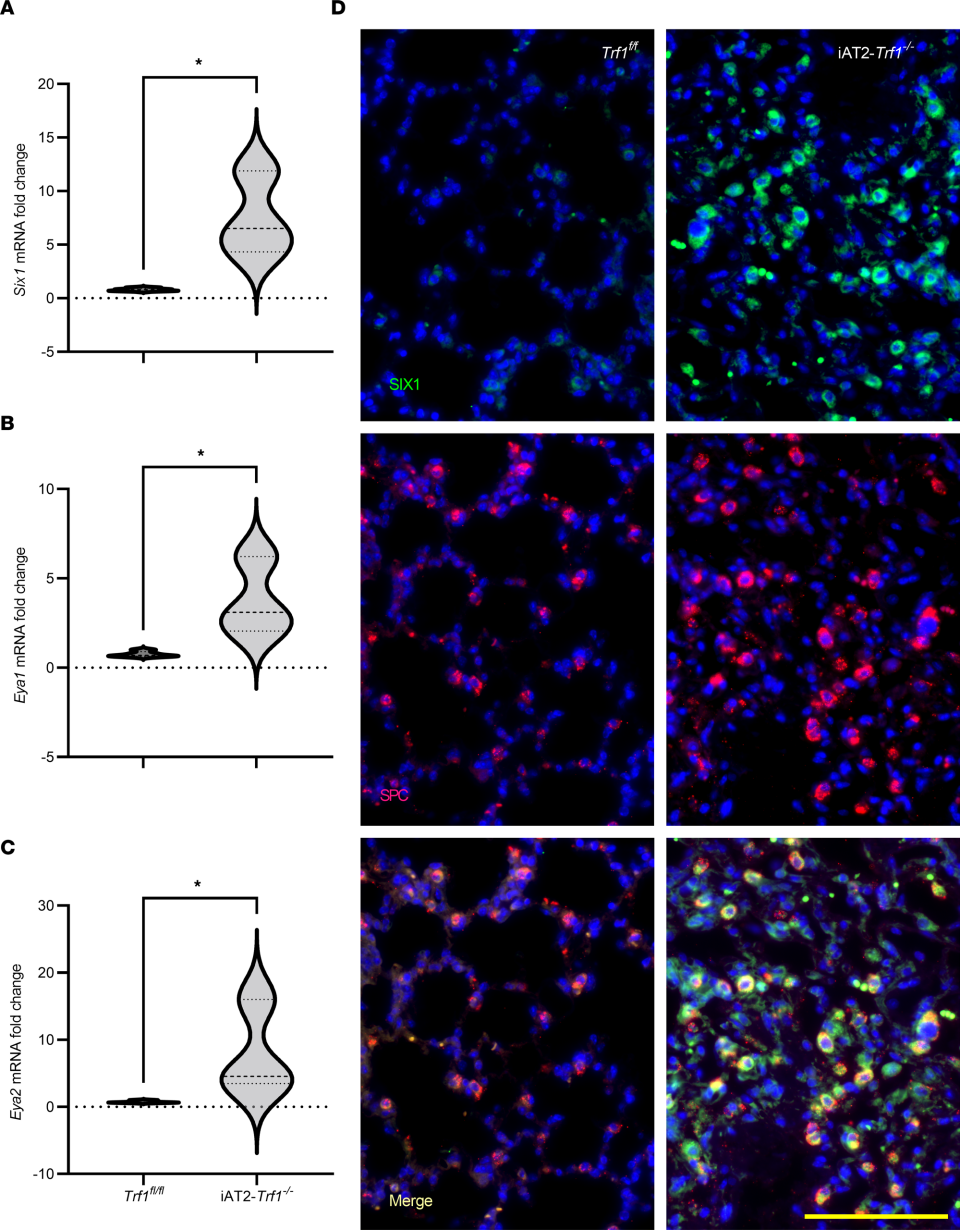
SIX1 levels are elevated in a model of spontaneous lung fibrosis. (**A**–**C**) *Six1* (**A**), *Eya1* (**B**), and *Eya2* (**C**) mRNA expression in AT2 cells isolated from *Trf1^fl/fl^* control mice or iAT2*^Trf11–/–^* mice. Levels of mRNA are represented as fold changes to *Trf1^fl/fl^*, set to 1-fold. *P ≤ 0.05 refers to 2-tailed, unpaired Student’s *t* test with Welch’s correction. (**D**) Representative immunofluorescence images of SIX1 (green signal) expression and the colocalization with SPC^+^ (red signal/merged) cells in lung tissue from *Trf1^fl/fl^* control and iAT2*^Trf1–/–^* mice. Scale bar: 100 μm.

BLM models do not recapitulate all features of human idiopathic interstitial lung diseases (24), and in recent years, genetic models of lung fibrosis have been explored (25). Recently, it has been shown that mice lacking the telomere shelterin protein telomere repeat binding factor 1 (TRF1) in AT2 cells develop spontaneous lung fibrosis after 9 months, thus providing a model that parallels what is known about telomere dysfunction in IPF (25). We determined levels of SIX1 in isolated AT2 cells from a conditional AT2-specific TRF1 (Spc-Cre *Trf1^fl/fl^*) mouse model. These studies revealed increased *Six1* (*7-fold*), *Eya1* (*3-fold*), and *Eya2* (5-fold) transcript levels in the isolated AT2 cells of these mice at 9 months after tamoxifen treatment (Figure 4, A–C). Immunofluorescence imaging of Spc-Cre *Trf1^fl/fl^* lung sections demonstrated increased SIX1 and colocalization with AT2 (SPC^+^) cells compared with *Trf1^fl/fl^* control mice (Figure 4D). Taken together, our results demonstrate that *Six1*, *Eya1*, and *Eya2* are increased in 2 distinct experimental models of fibrotic lung disease.

### Deletion of Six1 from AT2 cells protects mice from BLM-induced lung fibrosis.

**Figure 5:**
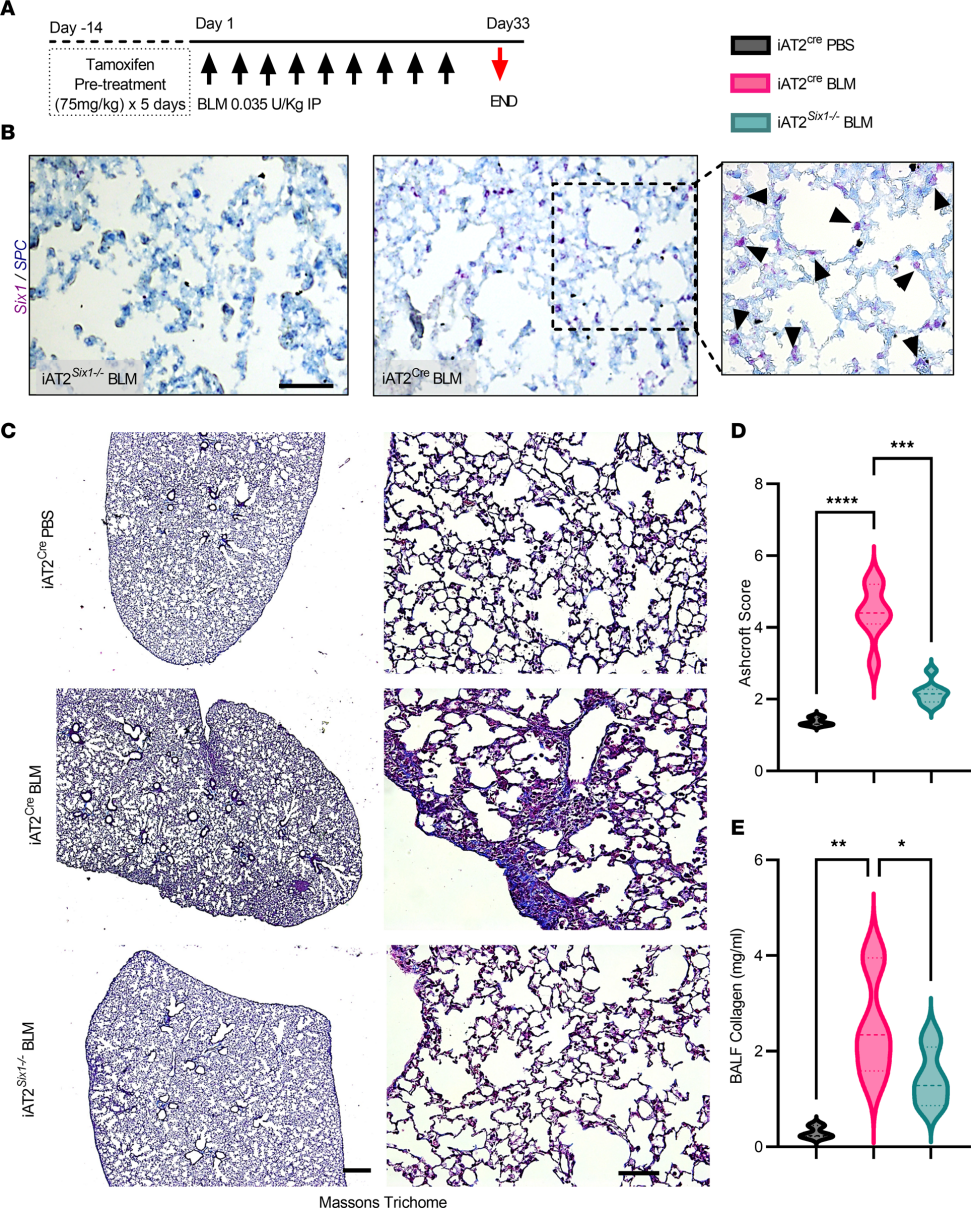
Prophylactic deletion of SIX1 prior to injury protects mice from lung fibrosis. (**A**) Experimental model with pretreatment of tamoxifen (75 mg/kg i.p. for 5 consecutive days) initiated 14 days prior to start of BLM or PBS treatment. (**B**) Representative RNA in situ hybridization images showing the absence of Six1 (72) signals in tamoxifen-treated, BLM-exposed iAT2*^Six1–/–^* and the presence of Six1 colocalized with SPC (blue) in tamoxifen-treated, BLM-exposed iAT2^Cre^ mice. Scale bar: 100 μm. Arrowheads depict *Six1* positive signals. (**C**) Representative Masson’s trichrome staining in low power field (left panels scale bar: 200 μm) and higher-power field (right panels scale bar: 100 μm) from iAT2^Cre^ mice treated with BLM or BPS and BLM-treated iAT2*^Six1–/–^* mice. (**D** and **E**) Ashcroft scores (*n =* 7) (**D**) and soluble collagen levels (mg/mL) from bronchoalveolar lavage fluid (BALF) (**E**) from PBS- or BLM-treated iAT2^Cre^ and iAT2*^Six1–/–^* (BLM only) mice (*n =* 5 per group). *P ≤ 0.05 and **P ≤ 0.01; ***P ≤ 0.001, ****P ≤ 0.0001 refer to Brown-Forsythe and Welch 1-way ANOVA tests with the 2-stage linear step-up procedure of Benjamini, Krieger, and Yekutieli post hoc test.

**Figure 6:**
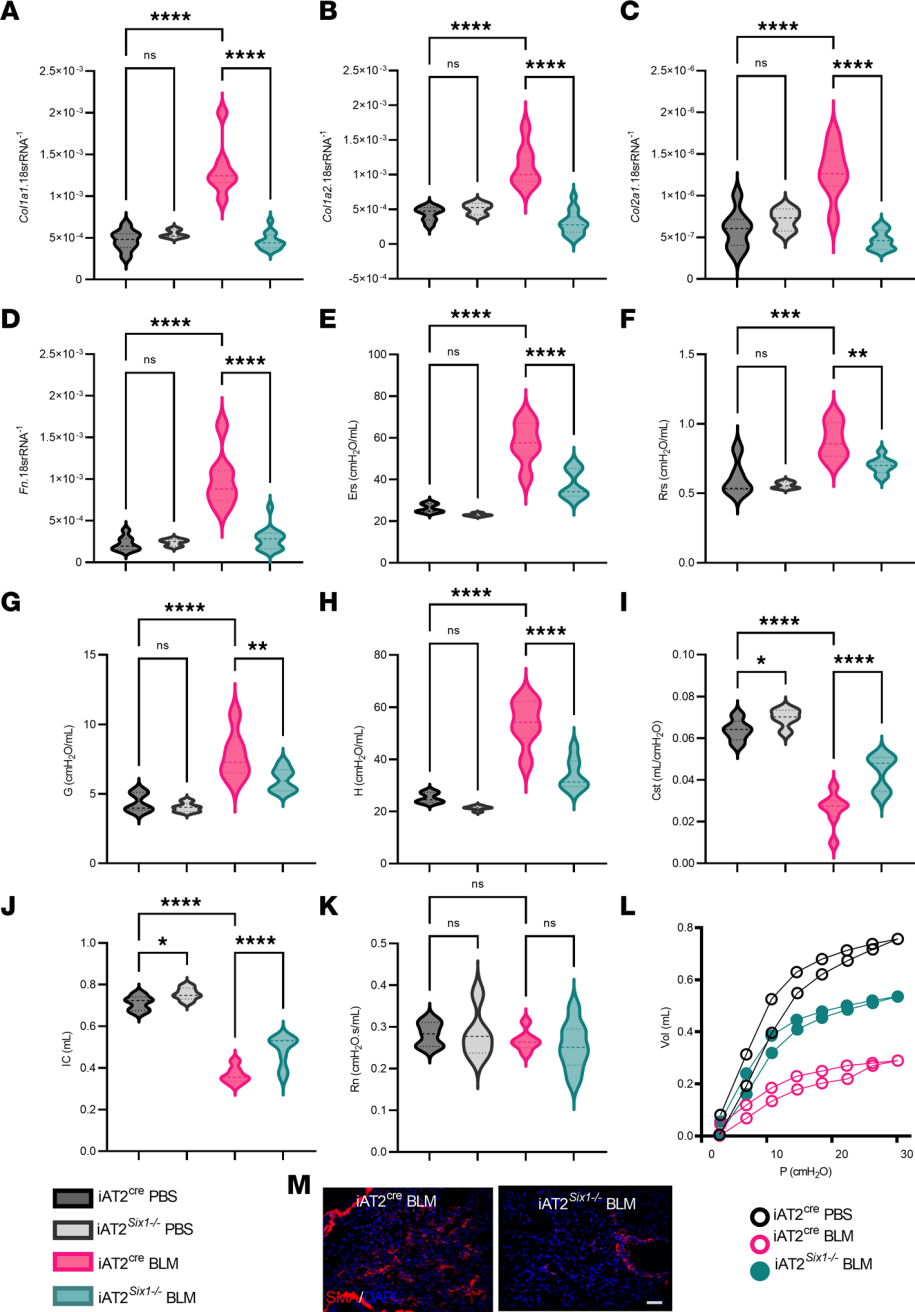
Fibrotic gene expression and lung function following prophylactic deletion of SIX1 in BLM-treated mice. (**A**–**D**) Transcript levels of for *Col1a1* (**A**), *Col1a2* (**B**), *Col2a1* (**C**), and *Fn* (**D**), from lung tissues isolated from BLM or PBS treated iAT2^Cre^ or iAT2*^Six1–/–^* mice. (**E**–**L**) Lung function data parameters for single frequency elastase (Ers, **E**), single frequency resistance (Rrs, **F**), tissue damping (G, **G**), tissue elastance (H, **H**), quasistatic compliance (Cst, **I**), inspiratory capacity (IC, **J**), Newtonian Resistance (Rn,**K**), and pressure-volume loops (**L**) from anesthetized PBS or BLM treated iAT2^Cre^ or iAT2*^Six1–/–^* mice (*n =* 5 for PBS treated mice, *n =* 6 for BLM-treated iAT2^Cre^ mice, and *n =* 8 for the BLM-iAT2*^Six1–/–^* group. *P ≤ 0.05, **P ≤ 0.01, ***P ≤ 0.001, ****P ≤ 0.0001 refer to 1-Way ANOVA tests with the 2-stage linear step-up procedure of Benjamini, Krieger, and Yekutieli post hoc test. (**M**) Immunofluorescence for α-smooth muscle actin (α-SMA, red) with DAPI counterstain from BLM treated iAT2^Cre^ or iAT2*^Six1–/–^* mice. Scale bar: 50 μm.

Embryonic deletion of *Six1* was shown to be lethal due to impaired epithelial branching, with mesenchymal hyperplasia leading to severe lung hypoplasia and respiratory failure (26). Thus, in order to evaluate whether *Six1* plays a pathophysiological role in lung fibrosis, we generated a tamoxifen-inducible conditional AT2-specific Six1-KO mouse (iAT2*^Six1–/–^)* using previously described SPC CreERT2 (27) and *Six1*-loxP/loxP mice (28). *Six1* was deleted prior to i.p. BLM exposure (Figure 5A). To confirm the deletion of *Six1* from AT2 cells following BLM treatment, we performed *Six1* RNA in situ hybridization with co-IHC with SPC (Figure 5B). These experiments demonstrated that mice lacking *Six1* in AT2 cells (iAT2^Six1–/–^) did not develop significant histological fibrosis compared with BLM-treated *Six1*-competent Cre-expressing control mice (iAT2^Cre^; Figure 5C), as assessed by Masson’s trichrome staining showing significantly reduced Ashcroft scores (Figure 5D). These changes were also consistent with reduced soluble collagen concentration in bronchoalveolar lavage fluid (BALF) of iAT2*^Six1–/–^* mice compared with control iAT2^Cre^ mice (Figure 5E) and consistent with attenuated levels of *Col1a1*, *Col1a2*, *Col2a1*, and *Fn* (Figure 6, A–D).

Disease progression in patients with IPF is often assessed using imaging and lung function analyses (29); thus, we performed lung function analyses (Figure 6, E–K). BLM-exposed iAT2^Six1–/–^ mice showed significant improvements in all measured parameters that increased in BLM-treated iAT2^Cre^ mice, including decreased whole respiratory elastance (Ers; Figure 6E) and whole respiratory resistance (Rrs, Figure 6F), parenchymal tissue damping (G, Figure 6G), and tissue elastance (H, Figure 6H) and increased inspiratory capacity (IC, Figure 6I) and static compliance (Cst, Figure 6J). Upper airway Newtonian resistance (Rn) showed no significant difference among all treatment groups (Figure 6K) and served as our internal control, since we did not anticipate any increase in the central/conducting airway resistance following BLM-induced fibrosis (22). The pressure-volume (PV) curve for BLM-treated iAT2*^Six1–/–^* mice still demonstrated a slightly more restrictive pattern compared with PBS controls (Figure 6L), but overall lung mechanics data appeared to be restored in SIX1-deficient mice compared with BLM-treated iAT2^Cre^ controls. We observed a significant reduction in the myofibroblast marker α-smooth muscle actin (α-SMA) in BLM-exposed iAT2*^Six1–/–^* compared with BLM-treated iAT2^Cre^ mice (Figure 6M). Taken together, these results demonstrate that prophylactic deletion of *Six1* prior to BLM exposure protects mice from the development of lung fibrosis observed both histologically and physiologically.

### Switching off Six1 during active fibrosis halts further lung injury.

**Figure 7:**
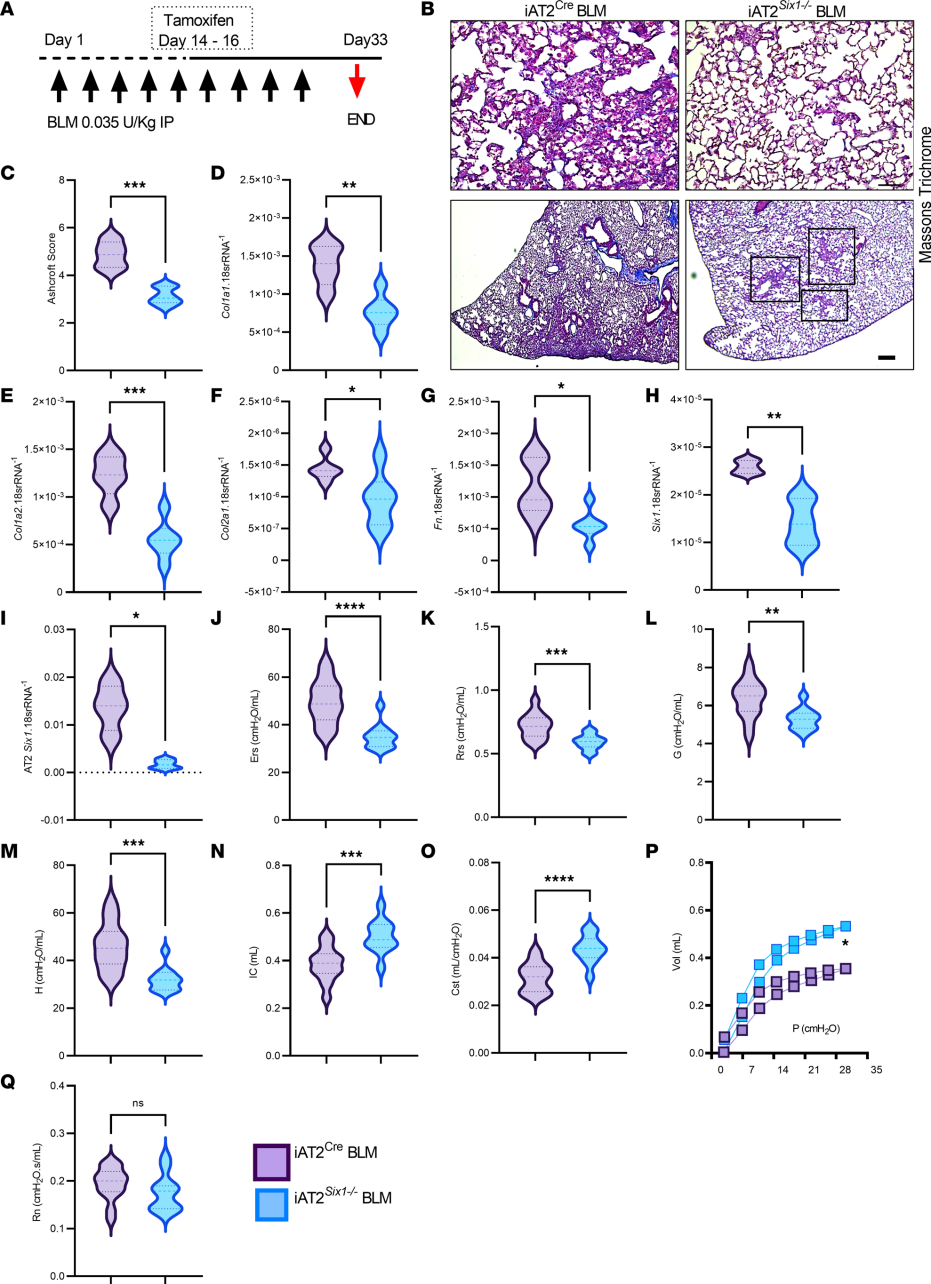
Deletion of SIX1 during active injury dampens lung fibrosis. (**A**) Experimental model where tamoxifen therapy started on day 15 of BLM treatment. (**B** and **C**) Representative Masson’s trichrome images at high-power field (top panels, scale bar: 100 μm) and low power field (bottom panels, scale bar: 200 μm) from BLM-treated iAT2^Cre^ or iAT2*^Six1–/–^* mice (**B**) and subsequent Ashcroft scores (**C**). (**D**–**G**) Gene expression levels of fibrotic markers *Col1a1* (**D**), *Col1a2* (**E**), *Col2a1* (**F**), and *Fn* (**G**). (**H** and **I**) qPCR levels for *Six1* from whole lung tissue (**H**) and isolated AT2 cells (**I**). (**J**–**Q**) Lung function data parameters for single frequency elastase (Ers, **J**), single frequency resistance (Rrs, **K**), tissue damping (G, **L**), tissue elastance (H, **M**), inspiratory capacity (IC, **N**), quasistatic compliance (Cst, **O**), pressure-volume loops (**P**), and Newtonian Resistance (Rn, **Q**) from anesthetized BLM treated iAT2^Cre^ or iAT2*^Six1–/–^* mice. *n =* 5 for **C**–**H**; *n =* 4 for **I**; *n =* 14 for **J**–**Q** for BLM-iAT2^Cre^ groups; *n =* 11 for panels **J**–**Q** in BLM-treated iAT2*^Six1–/–^* mice. *P ≤ 0.05, **P ≤ 0.01, ***P ≤ 0.001, ****P ≤ 0.0001 refer to Student’s 2-tailed *t* tests with Welch correction.

Next, we investigated whether switching off *Six1* during active fibrosis was able to halt fibrotic progression. In these experiments, we treated iAT2*^Six1–/–^* and iAT2^Cre^ mice with tamoxifen at day 15 after the initiation of BLM (Figure 7A). At this time point, evidence of fibrosis was already present in this model that included alterations in lung function, collagen deposition, and increased fibrotic gene expression as previously described (22). Excitingly, we observed a significant reduction in the histological burden of fibrosis (Figure 7, B and C), in line with reduced *Col1a1*, *Col1a2*, *Col2a1*, and *Fn* transcripts (Figure 7, D–G). To confirm deletion of *Six1* in these experiments, we measured transcript levels in lung tissue and in isolated AT2 cells from BLM-treated iAT2*^Six1–/–^* and iAT2^Cre^ mice, revealing reduced Six1 expression (Figure 7, H and I). These changes were consistent with in vivo lung function assessment that revealed significantly improved Ers (Figure 7J) and Rrs (Figure 7K) in BLM-iAT2*^Six1–/–^* compared with BLM-iAT2^Cre^ mice. We also report reduced lower airway tissue damping (G; Figure 7L) and tissue elastance (H; Figure 7M), increased IC (Figure 7N) and Cst (Figure 7O), an improved PV curve (Figure 7P), and no changes to Rn (Figure 7Q). Taken together, these results demonstrate that deletion of *Six1* during active injury attenuates the progression of ongoing lung fibrosis.

### AT2-specific SIX1 overexpression in vivo exacerbates lung fibrosis.

**Figure 8:**
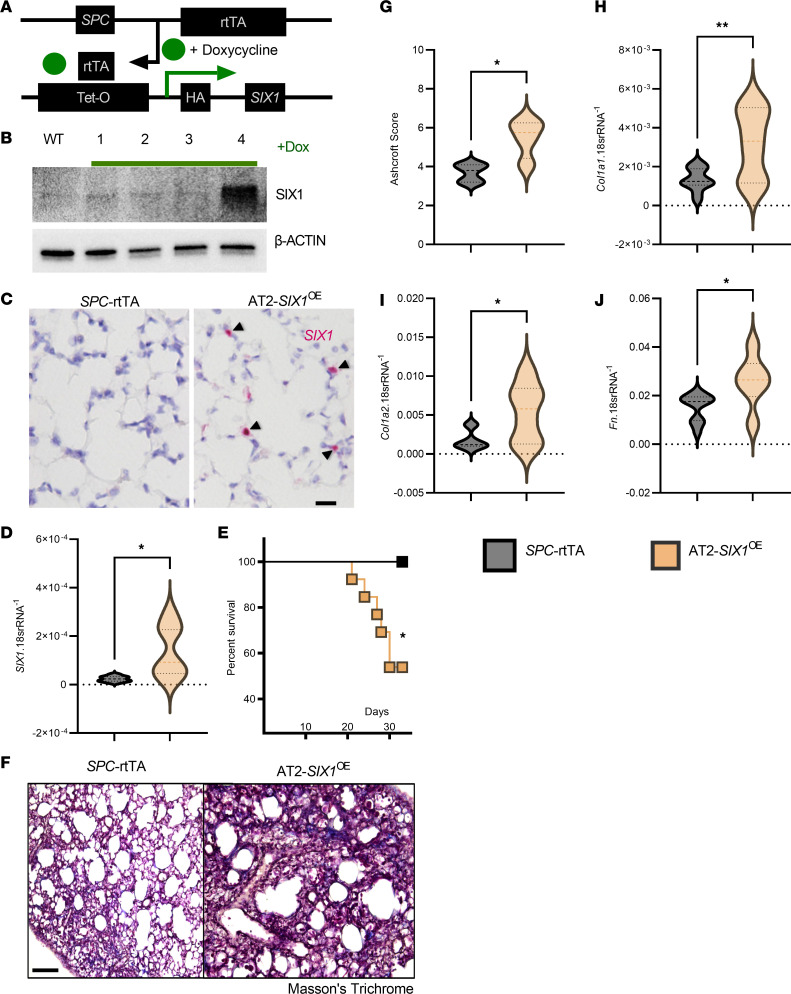
Overexpression of SIX1 in AT2 cells worsens lung fibrosis. (**A**) Representative model of the SIXTET;SPCrtTA (AT2-*SIX1*^OE^) genetics using the surfactant protein C (*SPC*) reverse tetracycline transcriptional activator (rtTA) driver under the control of doxycycline. (**B**) Western blot of isolated AT2 cells from wildtype C57BL/6 mice (WT) and the 4 possible genotypes used for the generation of the AT2-*SIX1*^OE^ mice treated with 14 days of 625 mg/kg doxycycline — in order from 1–4, respectively — from C57BL/6, AT2-*SIX1*^OE^ only, *SPC*-rtTA only, and AT2-*SIX1*^OE^. (**C**) RNA in situ hybridization of *SPC*-rtTA control mice and AT2-*SIX1*^OE^ showing expression of Six1 (pink) in AT2-*SIX1*^OE^ mice following doxycycline treatment. Scale bar: 20 μm. (**D**) Lung Six1 expression levels from BLM-treated *SPC*-rtTA and AT2-*SIX1*^OE^ mice. (**E**) Kaplan-Meier survival curve showing percent survival of AT2-*SIX1*^OE^ and *SPC*-rtTA challenged with i.p. BLM for 33 days. (**F**–**J**) Representative Masson’s trichrome stained lungs (**F**) (scale bar: 100 μm), Ashcroft scores (**G**) and qPCR for *Col1a1* (**H**), *Col1a2* (**I**), and *Fn* (**J**) from BLM-treated *SPC*-rtTA and AT2-*SIX1*^OE^ mice. *P ≤ 0.05 and **P ≤ 0.01 by Student’s *t* tests with Welch correction. *n* = 12 for all data panels except **G** (*n =* 7) for *SPC*-rtTA BLM groups; *n =* 7 for all data panels except **G** (*n =* 14) for BLM-*SIX*TET group.

We have demonstrated that deletion of *Six1* prophylactically or therapeutically prevents or halts the development of lung fibrosis. Next, we evaluated the effects of SIX1 overexpression in AT2 cells. To study this question, we generated an AT2 specific *SIX1* overexpression (AT2-*SIX1*^OE^) mouse using vector consisting of a human *SIX1* construct downstream of *tet* operator sequences (SIXTET) (30), with the *SPC* reverse tetracycline transcriptional activator (rtTA) driver (Figure 8A). Isolated AT2 cells from C57BL/6J, *SIX*TET only, *SPC*-rtTA only, or SIXTET;SPCrtTA (AT2-*SIX1*^OE^) at day 14 of doxycycline exposure revealed increased SIX1 protein expression in AT2-*SIX1*^OE^cells only (Figure 8B). This was consistent with increased *SIX1* RNA signals by RNA scope in *SIX*TET but not *SPC*-rtTA mice (Figure 8C) and by increased lung *SIX1* levels (Figure 8D).

BLM-treated AT2-*SIX1*^OE^ mice presented with a significant increase in mortality compared with *SPC*-rtTA mice (Figure 8E). Surviving mice presented with increased evidence of fibrosis histologically (Figure 8, F and G). This increased fibrotic burden observed histologically was in line with increased gene expression of *Col1a1*, *Col2a1*, and *Fn* and elevated Six1 levels in BLM-treated AT2-*SIX1*^OE^ mice compared with BLM-treated *SPC*-rtTA mice (Figure 8, H–J). These findings were consistent with in vivo lung function assessment that revealed a significant decline in lung function, including increased Ers and Rrs (Supplemental Figure 1, A and B; supplemental material available online with this article; https://doi.org/10.1172/jci.insight.142984DS1), increased lower airway tissue damping (G) and tissue elastance (H) (Supplemental Figure 1, C and D), and decreased IC and Cst (Supplemental Figure 1, E and F), in line with attenuated forced expiratory volume in 0.1 seconds (FEV_0.1_) and forced vital capacity (FVC) parameters (Supplemental Figure 1, G and H) in BLM-AT2-*SIX1*^OE^ compared with BLM-*SPC*-rtTA mice.

SIX1 expression is associated with increased proliferation of the cancer cells (30, 31). Thus, we aimed to study how Six1 affected proliferation in a noncancer lung cell model using the MLE12 mouse AT2 cell line. We used MLE12 cells overexpressing *Six1* (*Six1*OE) compared with GFP-expressing controls — as well as *Six1*-siRNA–treated cells, due to MLE12 cells having a low to moderate level of endogenous *Six1* expression — and verified *Six1* expression using qPCR (Supplemental Figure 2A). Surprisingly, *Six1*OE did not show significant changes in cell proliferation, as measured using the WST-1 assay (Supplemental Figure 2B). *Six1*OE also did not show significant changes in the quantification of phosphorylated histone H3 at serine 10 (pHH3) compared with the GFP control (Supplemental Figure 2, C and D) as a measure of cell proliferation. Interestingly, the siRNA-treated cells showed a significant increase in both WST-1 and pHH3 assays, suggesting higher active cell division and proliferation compared with *Six1*OE (Supplemental Figure 2, B–D). Although we expected *Six1* to promote cell proliferation, our results are in line with the decline in AT2 cells in IPF (32).

### SIX1 modulates macrophage migration inhibitory factor (MIF) in AT2 cells.

**Figure 9:**
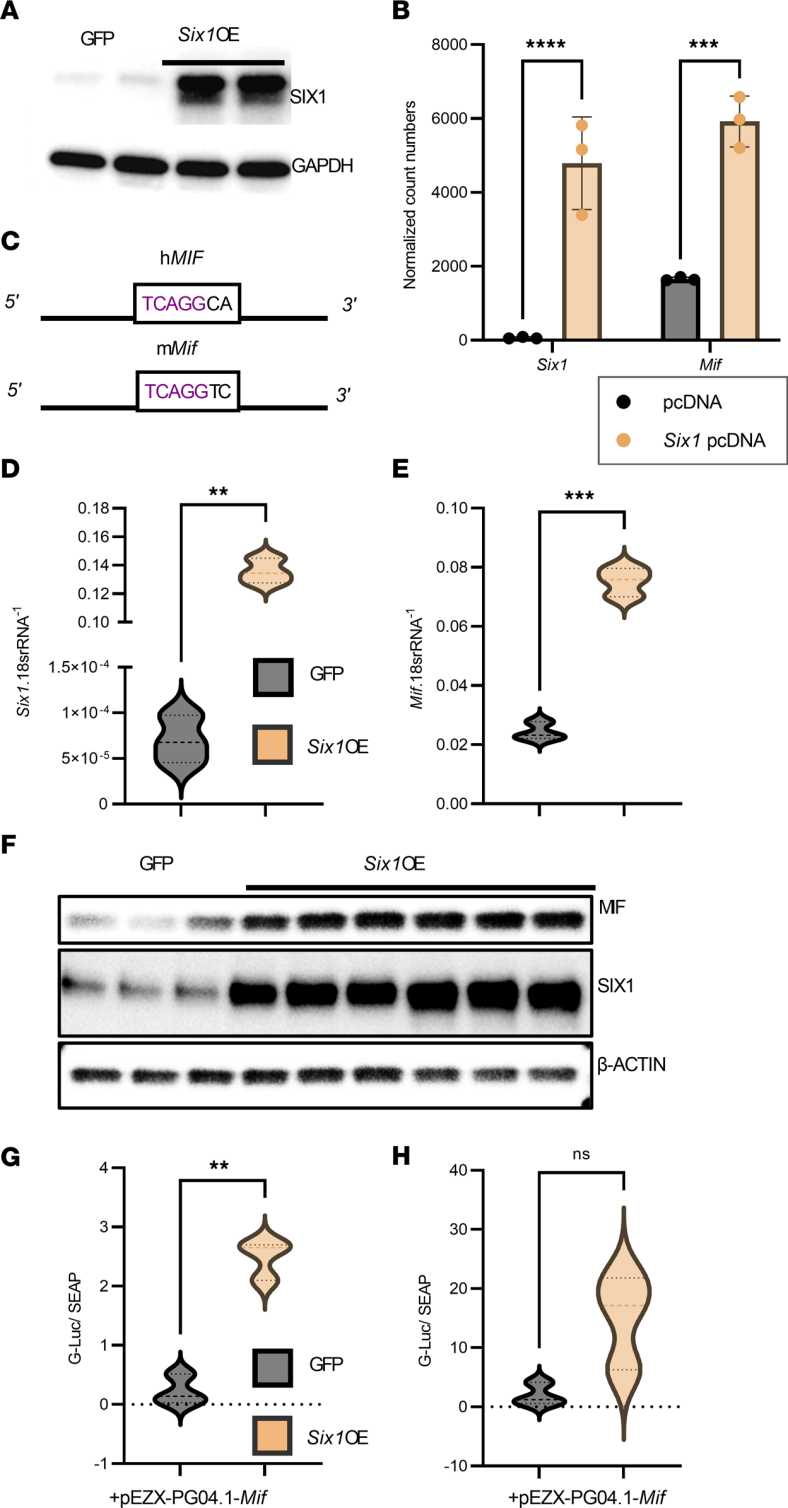
SIX1 overexpression upregulates MIF. (**A**) Western blot showing SIX1 protein overexpression using pCDNA-m*Six1* vector (*Six1*OE) in MLE-12 cells compared with GFP-encoding pCDNA control vector-transfected MLE12 cells. (**B**) RNA-Seq data normalized count numbers ± SD comparing GFP control MLE12 cells (*n =* 3) to *Six1*OE (*n =* 3); ***P ≤ 0.001 and ****P ≤ 0.0001 refer to 2-way ANOVA comparisons between pcDNA and *Six1*pcDNA with the 2-stage linear step-up procedure of Benjamini, Krieger, and Yekutieli post hoc test. (**C**) Representation depicting the *Six1* binding sequence (TCAGG) in the mouse and human *Mif* promoters. (**D** and **E**) qPCR showing increased expression of *Six1* (**D**) and *Mif* (**E**) in *Six1*OE cells. (**F**) Western blots showing increase in Six1 and MIF protein levels in *Six1*OE cells compared with GFP controls. (**G** and **H**) Ratios of Gaussian luciferase/secreted embryonic alkaline phosphatase (G-Luc/SEAP) showing levels of *Mif*-promoter activity from *Six1*OE cells compared with GFP controls at 12 hours (**G**) or 24 hours (**H**) of stimulation. **P ≤ 0.01 by Student’s *t* tests with Welch correction.

To identify how *Six1* expression promotes lung fibrosis, we overexpressed *Six1* in MLE12 cells (Figure 9A) and performed RNA-Seq (Supplemental Figure 3) to identify candidate genes.

Intriguingly, we found that MIF expression levels in *Six*OE were significantly increased compared with GFP controls (Figure 9B). *SIX1* has been shown to bind to several iterations of the *MIF* motif, and binding to the consensus pentanucleotide sequence 5′-TCAGG-3′ was critical, while other positions were able to change bases with similar dose kinetics (33, 34). We examined the promoter sequences of both human and mouse *MIF* genes and found both of them to contain 5′-TCAGG-3′ and to be part of the *Mif*-Luc promoter plasmids (Figure 9C). To further test if *Six1* could modulate *Mif* levels and validate our RNA-Seq, we measured *Mif* transcript levels by qPCR and confirmed increased levels of *Mif* in *Six1*OE cells compared with GFP controls (Figure 9, D and E). These results were consistent with increased MIF protein levels in the *Six1*OE cells consistent with increased SIX1 (Figure 9F). We further confirmed *Mif* promoter binding by Six1 using a full-length *Mif* promoter Gaussia Luciferase–expressing plasmid, wherein luciferase activity was significantly increased in *Six1*OE within 12 hours after transfection but was no longer significant at 24 hours (Figure 9, G and H).

**Figure 10:**
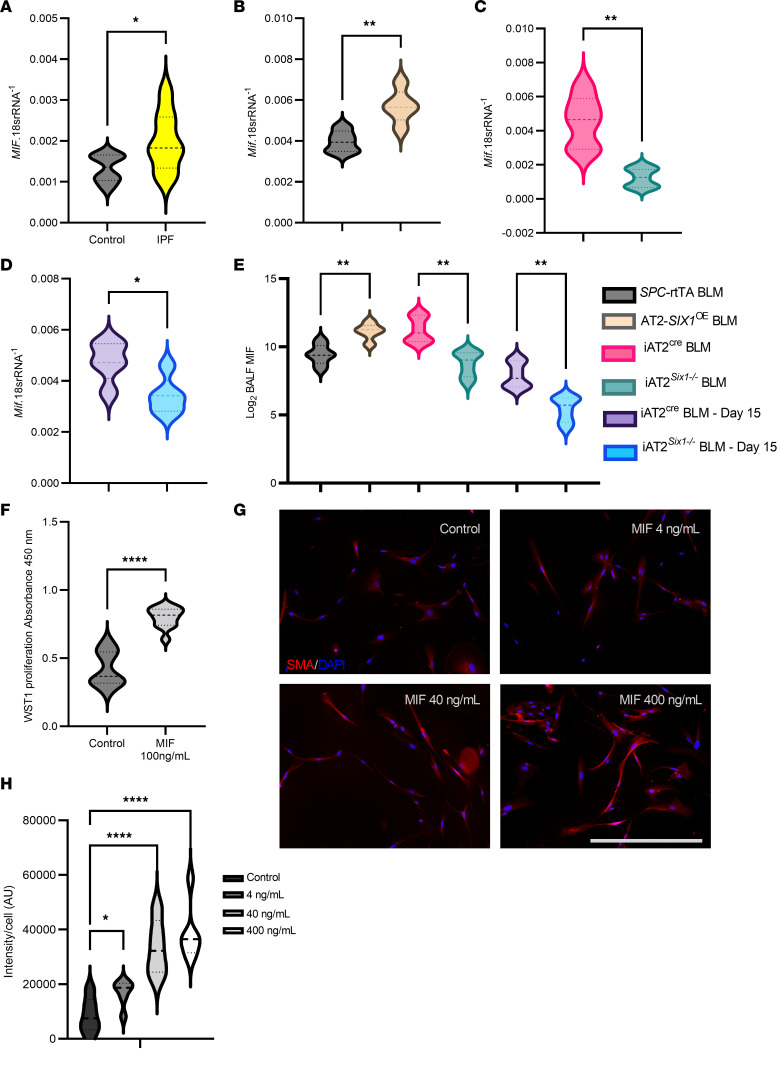
Elevated MIF levels in IPF and in experimental models of lung fibrosis. (**A**) *MIF* transcript levels in control or IPF lung samples (*n =* 8 per group). (**B**–**D**) Lung *Mif* transcript levels from BLM treated *SPC*-rtTA or AT2-*SIX1*^OE^ mice (*n =* 5 per group) (**B**); BLM-treated iAT2^Cre^ or iAT2*^Six1–/–^* treated prophylactically (*n =* 6 [iAT2^Cre^], *n =* 7 [iAT2 *^Six1–/–^* ]) (**C**) or therapeutically, starting on day 15 of BLM (*n =* 5 per group) (**D**) with tamoxifen to delete AT2-*Six1*. (**E**) MIF BALF levels from BLM treated *SPC*-rtTA or AT2-*SIX1*^OE^ mice; BLM-treated iAT2^Cre^ or iAT2*^Six1–/–^* treated prophylactically or therapeutically with tamoxifen. (**F**) Absorbance values of WST-1 assay at 24 hours read at 450 nm for control human lung fibroblasts (*n =* 12) with or without 100 ng/mL recombinant human MIF. (**G**) Human lung fibroblasts treated with a dose response (4–400 ng/mL) of MIF in vitro for 48 hours stained with α-SMA (red signal) and counter-stained with DAPI. Scale bar: 50 μm. (**H**) Quantification of SMA fluorescent signal using integration of per cell fluorescence pixel intensity using an automated fluorescence cell cytometer (*n =* 5 per group). *P ≤ 0.05, **P ≤ 0.01, ****P ≤ 0.0001 refer to Kruskal-Wallis test with the 2-stage linear step-up procedure of Benjamini, Krieger, and Yekutieli post hoc test (**A**), Student’s *t* test with Welch Correction (**B**–**D**, and **F**), and Browne-Forsythe and Welch ANOVA tests with Dunnett’s T3 multiple comparison test (**E** and **H**).

In IPF, prior studies have shown an increase in MIF concentration in the BALF and increased expression in lung tissue from patients with IPF compared with control samples (35, 36). Consistent with these reports, we demonstrate that *MIF* transcript levels are increased in IPF lungs compared with control samples (Figure 10A). We observed that overexpression of *SIX1* in AT2 cells using the AT2-*SIX1*^OE^ mice resulted in increased expression of *Mif* transcripts compared with BLM-treated *SPC*-rtTA controls (Figure 10B). Similarly, prophylactic or therapeutic deletion of *Six1* (starting on Day 15 of BLM) resulted in reduced *Mif* lung expression in BLM-treated iAT2*^Six1–/–^* mice compared with the BLM-treated iAT2^Cre^ groups (Figure 10, C and D). Consistent with our transcript data, MIF BALF levels were upregulated in BLM-exposed AT2-*SIX1*^OE^ mice, but were reduced in AT2-SIX*^–/–^* following prophylactic or therapeutic deletion of *Six1* (Figure 10E).

**Figure 11:**
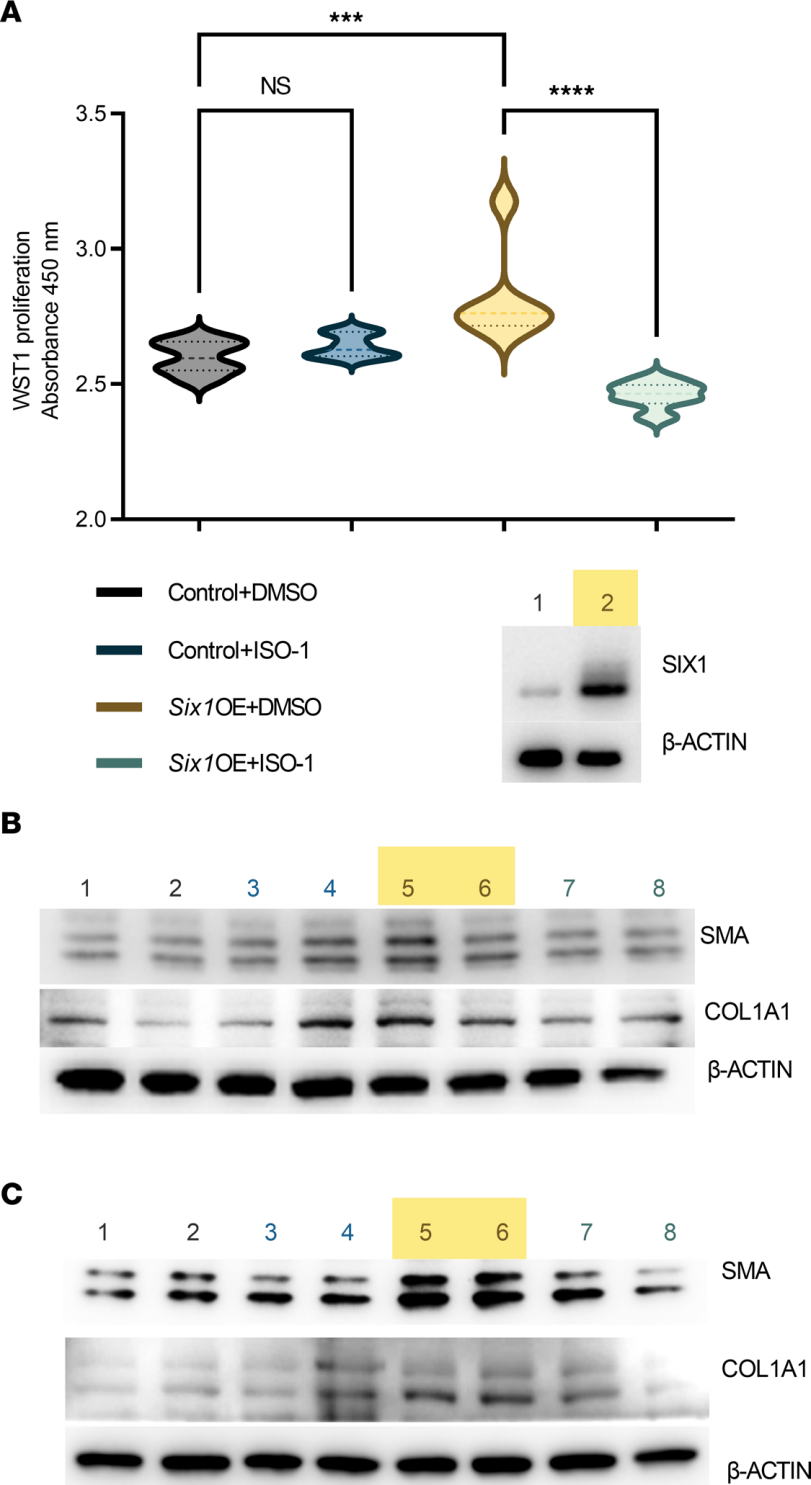
Effects of SIX1 overexpression are blocked by a MIF inhibitor. (**A**) WST-1 proliferation assay from GFP-encoding pCDNA (control), SIX1 protein overexpression using pCDNA-m*Six1* vector (Six1OE) transfected MLE-12 cells cocultured in transwells treated with ISO-1 (a MIF inhibitor), or DMSO (vehicle for ISO-1). Western blot depicts successful SIX1 expression from pCDNA-m*Six1* (lane 2) compared with control (lane 1) transfected MLE-12 cells. ***P ≤ 0.001, ****P ≤ 0.0001 refer to 1-way ANOVAs with the 2-stage linear step-up procedure of Benjamini, Krieger, and Yekutieli; *n =* 6 for all groups. (**B** and **C**) Western blots for smooth muscle actin (SMA), Collagen 1a1 (COL1A1), and β-actin from coculture studies using transwell assays (**B**) or conditioned media (**C**). Lanes 1 and 2 represent control treated cells (GFP-encoding pCDNA) + DMSO; lanes 3 and 4 represent control treated cells (GFP-encoding pCDNA) + ISO-1; lanes 5 and 6 represent *Six1* OE cells (pCDNA-m*Six1* vector) + DMSO; and lanes 7 and 8 represent Six1 OE cells (pCDNA-mSix1 vector) + ISO-1.

MIF has also been shown to affect the proliferation of neighboring fibroblasts in cardiac tissues (37) and dermal fibroblasts (38), but how secreted MIF affects lung fibroblasts remains poorly understood. We explored how direct exposure to MIF protein in vitro affected primary human lung fibroblasts by using isolated fibroblasts from 4 independent control donors and exposing them to a known concentration of recombinant human MIF for 24 hours. We found that human lung fibroblasts treated with 100 ng/mL of recombinant human MIF increased fibroblast cell proliferation as measured by WST-1 proliferation assay (Figure 10F). We then exposed fibroblasts to a dose response (4–400 ng/mL) of MIF in vitro for 48 hours to assess for changes to fibroblast differentiation using α-SMA as a marker. We demonstrated no significant change at the lower 4 ng/mL exposure to MIF, but we show a significant dose-dependent increase from baseline with both the 40 and 400 ng/mL doses as assessed by the integration of per cell fluorescence pixel intensity using an automated fluorescence cell cytometer (Figure 10, G and H). Next, we aimed to test whether the MIF inhibitor ISO-1 (39) was able to inhibit the profibrotic effects of *Six1* overexpression. MLE12-overexpressing *Six1* were cultured directly with mouse 3T3 fibroblasts (3T3) (transwell) or indirectly (MLE12 conditioned media on 3T3 cells); we demonstrate that cell proliferation and α-SMA and COL1A1 expression levels are attenuated by the MIF inhibitor, ISO-1 (Figure 11, A–C).

## Discussion

AT2 cells are shown to have significant epithelial plasticity to allow for repair of the injured alveolar space, and in IPF, they have been shown to inappropriately express developmental gene pathways (11, 40, 41). Despite this observation, only a few studies have interrogated the significance of reactivated developmental pathways. In this study, we focused on the critical developmental gene *SIX1* and its function in AT2 cells. We tested our hypothesis that SIX1 is a molecular driver of lung fibrosis that can be targeted therapeutically.

SIX1 is an essential transcription factor for normal lung morphogenesis in utero (13) and is normally not expressed at significant levels in most adult tissues. Absence of SIX1 in mice leads to severe epithelial branching abnormalities and pulmonary hypoplasia, underscoring the vital role SIX1 plays in normal lung development (13, 26). In contrast, various adult pathologies demonstrate inappropriately increased SIX1 expression in adult tissues, including breast, esophageal, and lung cancers (16, 42, 43). Although SIX1 has not been previously studied in lung fibrosis, an ovalbumin mouse model of asthma and a bronchial epithelial cell line (16HBE) showed that RNAi knockdown of *Six1* decreased OVA-challenged inflammation, infiltration, and mucus production and that *SIX1* siRNA-treated 16HBE cells suppressed TGF-β1–mediated epithelial-to-mesenchymal transition with a decrease in fibronectin and collagen IV expression (44, 45). In this study, we demonstrate that there is increased SIX1 transcript and protein expression, and increased transcript and protein expression of its cofactors EYA1 and EYA2, in IPF compared with controls; we also demonstrate that conditional deletion of *Six1* and overexpression in AT2 cells in mice can significantly attenuate or exacerbate lung fibrosis, respectively. Although several big data studies have identified increased levels of *SIX1* in both lung tissue (18, 19), and in AT2 scRNA-Seq data (20) from IPF lungs, its mechanistic role has not been studied until now. It is also important to note that, although landmark studies developing the IPF lung atlas do not appear to show increased *SIX1* in AT2 cells, increased *SIX1* is observed in SPC^+^ cells — in particular, aberrant basaloid cells, a unique population within IPF lungs (46–50). Thus, it is conceivable that SIX1 may play an important role in transformed epithelial cells such as aberrant basaloid cells, which can contribute to the pathophysiology of IPF (46). It is important to note that, although our study focuses on the role of SIX1 on AT2 cells, where we report increased levels, elevated *SIX1* expression was also observed in mesenchymal and bronchial epithelial cells (Supplemental Figure 4). Thus, it is possible for SIX1 to have important profibrotic functions in other cells.

MIF is a conserved 12.5 kDa cytokine that has been shown to signal through the binding of cell-surface receptors CXCR2, CXCR4, CD74, and CXCR7 (51, 52). MIF is a proinflammatory and profibrotic cytokine that has been shown to be increased in several chronic lung diseases including IPF, pulmonary arterial hypertension (PAH), and asthma and in fibrotic disorders including skin fibrosis of patients with both limited systemic sclerosis (lSSc) and diffuse systemic sclerosis (dSSc), as well as renal and cardiac fibrosis (35, 53, 54). In IPF, studies have shown an increase in MIF concentration in the BALF and increased expression in lung tissue from patients with IPF compared with control samples. Specifically, MIF expression in tissue was localized to the alveolar epithelium and fibroblasts in areas of active fibrosis (35, 36). More importantly, recent studies in the BLM model of lung fibrosis revealed that treatment with an anti-MIF antibody showed decreased mortality, lung injury, and significantly lower inflammation but now lower collagen content at day 21, whereas mice treated with a small molecule, cell-permeable MIF antagonist (ISO-1) showed decreased fibrosis, collagen content, and vascular smooth muscle proliferation in BLM-treated mice compared with untreated controls (55, 56). This is an important difference, as anti-MIF antibodies can target secreted or systemic MIF without affecting MIF’s intracellular interaction with other signaling molecules, whereas non-protein-based inhibitors have the potential to affect both intracellular and secreted MIF pathways, both of which are still being actively investigated in chronic lung disease (53).

Similarly, there have been several studies using mouse or rat models of asthma that showed increase in MIF with increased Th-2 cytokines and IgE levels, airway hyperresponsiveness, and airway smooth muscle thickness (39, 57–61). When 2 of these studies (57, 60) used anti-MIF antibodies, researchers observed decreased features of asthma but did not see the decrease in Th-2 cytokines and IgE levels; however, when *Mif* was genetically deficient (61) or when using a nonantibody MIF inhibitor like ISO-1 (39), the Th-2 cytokines and IgE were significantly reduced. The role of MIF in IPF is not fully understood and how it is transcriptionally regulated has not been fully investigated. Herein, we have demonstrated a potentially novel mechanism whereby SIX1 modulates MIF expression by binding to the consensus sequence in AT2 cells that is important for the progression of lung fibrosis, and we demonstrated that AT2-secreted MIF contributes to downstream fibroblast activation and fibrosis development. Using coculture approaches with mouse fibroblast (3T3) and alveolar cells murine lung epithelial-12 (MLE12), we demonstrate that the SIX1-mediated effects on cell proliferation and expression levels of α-SMA and Col1a1 are reduced by ISO-1, a MIF inhibitor, providing further evidence for the SIX1-MIF link in lung fibrosis. Further understanding of the pleiotropic role of MIF on various cell types in the lung could lead to new mechanistic insights and novel treatment strategies. Taken together, these studies provide evidence that the role of MIF is complex. Intracellular regulation or targeting of MIF can provide additional benefits compared with anti-MIF antibodies alone and point at a profibrotic role for MIF. Our study proposes a potentially novel SIX1-MIF signaling axis where upregulation of SIX1 leads to both elevated intracellular and secreted MIF levels that can promote fibrosis.

One of the most challenging aspects of drug discovery for IPF is the identification of new targets using preclinical animal models of lung fibrosis that can be translated effectively (22, 62, 63), and this highlights the importance of our results, which show reduced progression of fibrosis and improved physiological lung function using the therapeutic deletion of Six1. Furthermore, the development of therapeutics that target transcription factors is challenging. However, recently, the crystal structure for the SIX1/EYA2 complex was determined, which showed that Six1 uses a single α helix to interact with EYA2 (64). This interaction is very similar to the protein-to-protein interactions of p53/MDM2 (65) and BAK/BCL-XL (66), which have been successfully targeted with small-molecule inhibitors. More importantly, the fact that a single amino acid substitution in this helix could sufficiently abrogate the SIX1-EYA2 interaction, targeting SIX1, may therapeutically mitigate transactivation of fibrosis-related pathways. In summary, IPF is an insidious, devastating disease with high mortality and limited therapeutic options. Our findings support a strong basis for the continued work on SIX1 in the alveolar epithelium and have the potential to lead to many insights into the molecular mechanisms of pulmonary fibrosis.

## Methods

### Processing human lung tissues and isolation of AT2 cells.

Deidentified lung explant tissue from patients with IPF and COPD with corresponding deidentified clinical parameters were obtained from the Houston Methodist Hospital. IPF or COPD was diagnosed by board-certified pulmonologists upon admittance for lung transplantation at the Houston Methodist Hospital. Control lung tissue was obtained from lungs that were declined for transplantation but had no chronic pulmonary disease or contusions. Healthy control lung tissue was obtained from the International Institute for the Advancement of Medicine (Edison, New Jersey, USA). The demographic details of the study population are summarized in Supplemental Table 1.

Tissue was collected at the time of lung explantation and processed on site within 20 minutes. The lobes were identified from each lung explant, and a piece from each portion was then cut for histology; an adjacent section was flash frozen in liquid nitrogen. Isolation of primary human AT2 cells was adapted from a previously described protocol (67), with the following modifications. After perfusion, lung tissue was minced, followed by digestion with 0.25% trypsin (MilliporeSigma). The tissue suspension was filtered twice through 100 μm and 40 μm cell strainers (Thermo Fisher Scientific). The obtained cell suspension was incubated in DMEM/F12 to remove macrophages and fibroblasts. The remaining cell solution was separated on a Percoll (MilliporeSigma) gradient. The obtained cells were plated for AT2 marker evaluation or processed for subsequent analysis.

### Animals.

Mice generated for this study included the development of an AT2-specific conditional KO mouse (iAT2^Six1–/–^) using the tamoxifen-inducible Cre recombinase (Cre-ERT2) under the control of the human SPC promoter as previously described (27) and *Six1*-loxP/loxP mice (in-house) (28). Additional mice generated included the doxycycline-inducible AT2 *SIX1* overexpressing AT2-*SIX1*^OE^ mice using *SIX1*Tet mice consisting of a human *SIX1* construct downstream of tet operator sequences as described (30) with the *SPC*-rtTA driver (B6.Cg-Tg [*SPC*-rtTA] 5Jaw/J; 006235, The Jackson Laboratory). To induce lung fibrosis, mice were administered 0.035U/g i.p. of BLM (Hospira Pharmaceuticals) or vehicle (PBS; Invitrogen) twice a week for 4 weeks,as previously described (22).

### Isolation of murine AT2 cells.

We used a previously described protocol (68) with modifications. Murine AT2 cells were isolated by dispersion of lungs with dispase and negative selection with antibodies against CD32/CD16 and CD45, followed by lamellar body staining with Lysotracker DND-26 (Cell Signaling Technologies [CST]) and pro-SPC polyclonal antibody (MilliporeSigma) to stain SPC.

### Isolation of human lung fibroblasts.

Tissue from healthy human lung explants was collected from anatomically comparable regions. After tissue perfusion, approximately 2–4 fragments 10 mm^3^ were minced and subsequently digested in DMEM supplemented with 0.25% trypsin for 1 hour at 37°C. The tissue suspension was filtered through large mesh followed by 100 μm cell strainers (Thermo Fisher Scientific). The obtained cell suspension was incubated in DMEM/F12 to allow fibroblasts to adhere. Cells were then washed to remove the enzyme and cultured in DMEM supplemented with 10% FCS and penicillin/streptomycin for 1 week to allow fibroblasts to proliferate and become the dominant cell type. Cultured fibroblasts were used for experiments between passages 1 and 3.

### Lung function assessment.

We performed lung function measurements as previously described (22) with the following modifications. We used the FlexiVent FX system (SCIREQ Inc.) equipped with the FX1 module and Flexiware v8.3 software. On day 33, mice were anesthetized with an i.p. injection of 5% Avertin (0.012 mL/g body weight). A tracheostomy was performed and trachea cannulated with an 18-gauge metal cannula with a resistance of approximately 0.2–0.3 cmH_2_O·s/mL. This was followed by the generation of a PV curve using a ramp-style pressure-driven (PVr-P) maneuver to a maximum of 30 cmH_2_O. The following are the read-outs included in our study: Ers and Rrs measurements using the single frequency flexiVent forced oscillation (69) perturbation under closed chest conditions; partitioned, broadband FOT showing tissue damping (G) and tissue elastance (H); IC with Cst and PVr-P maneuver; and Rn of central airways.

### Cell culture and plasmids.

Mouse alveolar lung epithelial cell line MLE12 (ATCC-CRL-2110, ATCC; ref. 70) was used to assess Six1 function in vitro. Cells were cultured using DMEM/F12 (Thermo Fisher Scientific, 12634010) with 2% FBS supplemented with 0.005 mg/mL insulin, 0.01 mg/mL transferrin, 30 nM sodium selenite (Corning, ITS 354350), 10 nM hydrocortisone (Sigma H6909), 10 mM β-estradiol (Sigma E2758), 10 mM HEPES buffer (Corning 25-060-CI), and 2 mM L-glutamine (HITES) (Sigma 59202C). Plasmids used include Six1-pcDNA3.1 and Flag-Eya2-pcDNA3.1 (Addgene, 49264). The generation of the pcDNA3.1-SIX1 plasmid included the subcloning of the full-length human or mouse *Six1* cDNA (in-house) into the pcDNA3.1 backbone using the Xho1 and EcoR1 restriction sites.

### MIF-Luc promoter binding assay.

Mouse lung epithelial cells were cotransfected with either control pcDNA-GFP vector or a pcDNA-mSix1 construct made in our laboratory, along with the pEZX-mMIFpromoter–Gaussia Luciferase expression plasmid (pEZX-PG04.1; MPRM41428-PG04, GeneCopoeia). Supernatants were collected at 12, 24, and 48 hours after transfection and assessed for secreted luciferase activity, along with secreted embryonic alkaline phosphatase, as the reference control using Secrete-Pair dual luminescence assay kit (GeneCopoeia, LF032). Experiments were run in triplicate wells for all the experimental groups, and supernatant samples that were collected at various time points were flash frozen until the analysis.

### MIF recombinant protein, ELISA, WST-1 assay, and image analysis.

To detect secreted MIF protein levels, we used the MIF in vitro SimpleStep ELISA (Abcam, 209885) assay per manufacturer’s protocol with the following modifications. BALF was diluted 1:100 in sterile PBS for all samples. Human recombinant MIF (MilliporeSigma, SRP3321) was used for all experiments involving primary human lung fibroblasts. WST-1 assay (Roche, 05015944001) was performed according to manufacturer’s protocol. Briefly, 6 × 10^3^ fibroblasts were seeded in 96-well plates, serum starved overnight, and then supplemented with DMEM with 10% FBS and 100 ng/mL MIF for 24 hours. In total, 10 μL/well of WST-1 reagent was added and the absorbance was read at 450 nm after 4 hours. Image analysis of α-SMA (Abcam, ab5694) was performed using the BZ-X800 high-resolution automated fluorescence microscope and image analysis system (Keyence), using the image cytometry analysis software with brightness (integration) mode for the automated analysis of fluorescence intensity per cell under standardized conditions. For all groups, > 500 cells were analyzed.

### Coculture experiments.

Mouse-derived 3T3 fibroblasts (ATCC-CRL-1658, ATCC) were plated in 12-well tissue cultured plates, and MLE12 cells were plated in transwell inserts with a 3 μM pores in a separate plate. MLE12 cells were then transfected with Six1-pcDNA3 overexpression vector or control GFP-pcDNA3 vector at 1 μg/well with jetPRIME (Polyplus-transfection). After 24 hours, the MLE12 cells were washed with phosphate buffered saline (PBS) and fresh media added with ISO-1 (Bio-Techne, 4288) at 50 μg/mL or DMSO control; then, the transwell inserts were placed in the plates with the 3T3 cells. After 48 hours, both 3T3 and MLE12 cells were harvested for protein and separate 3T3 wells were assayed by WST assay (MilliporeSigma, 11465007001).

Mouse 3T3 fibroblast were plated in 12-well plates, and separate plates were seeded with MLE12 cells. MLE12 cells were transfected with Six1-pcDNA3 overexpression vector or control GFP-pcDNA3 vector at 1 μg/well with jetPRIME. After 6 hours, the MLE12 cells were washed and fresh media was added. At 24-hour increments, media was moved from the MLE12 cells, centrifuged to remove debris at 1000 *g at* 4° C for 5 minutes, and added to the 3T3 cells to refresh with the addition of 50 μg/mL ISO-1 or DMSO control. After 72 hours, the cells were harvested for protein.

### RNA-Seq.

MLE12 cells were transfected with pcDNA3.1-Six1 using jetPRIME per the manufacturer’s protocol. Cells were collected and lysed using TRIzol (Invitrogen), and total RNA was purified using the RNeasy Kit (QIAGEN). Total RNA-Seq was performed with MiSeq (LC Sciences). RNA-Seq reads were aligned to the mouse genome (GRCm38, mm10) using Gencode VM23.

Full RNA-Seq data are available with the accession no. PRJNA824067 (https://www.ncbi.nlm.nih.gov/bioproject/PRJNA824067).

### RNA in situ hybridization.

The following protocol was designed using the validated hybridization probes that target the following sequences: mouse SIX1 (1932–2819 of NM_009189.3) and human SIX1 (799–2177 of NM_005982.3). We followed Advanced Cell Diagnostics (ACD) manufacturer protocol for RNA-ISH (RNAScope, ACD). Briefly, slides were deparaffinized through xylene and 100% ethyl alcohol, and they were quenched with hydrogen peroxide for 10 minutes at room temperature. Slides were then washed in distilled water and placed in slow boiling Target Retrieval Reagent (RNAScope Target Retrieval, ACD) for 15 minutes. Slides were incubated with protease treatment (RNAScope Protease Plus, ACD) for 30 minutes in a humidifying oven at 40°C. After protease treatment, slides were washed in distilled water and incubated with RNAScope probe for 2 hours in a 40°C humidifying oven. Between each incubation step, slides were washed in wash buffer (RNAScope Wash Buffer, ACD) twice for 2 minutes each with agitation. Slides were probed with AMPs 1-6 (ACD) as per the manufacturer’s instructions. Detection reagents (RNAScope 2.5 Detection Kit RED, ACD) were added for 10 minutes and were counterstained with hematoxylin. Slides were cleared in xylene and immediately mounted with nonaqueous sealant (CytoSeal, Thermo Fisher Scientific).

### Histological and gene expression analyses.

On day 33, histological analysis and gene expression quantifications were performed as previously described (71) using the antibodies and primers listed in Supplemental Table 2 and Supplemental Table 3.

### Statistics.

Prism software (v9.0; GraphPad or higher) was used for all statistical analyses. Student’s *t* test (2-tailed) with Welch’s correction / Mann-Whitney *U* test was used to compare any paired conditions. One-way ANOVA (72), followed by the 2-stage linear step-up procedure of Benjamini, Krieger, and Yekutieli post hoc test, was used when multiple conditions were being compared. *P* ≤ 0.05 was considered significant.

### Study approval.

The use of human material was reviewed and approved by the UTHealth Committee for the Protection of Human Subjects (HSC-MS-08-0354 and HSC-MS-15-1049). All studies utilizing animals were reviewed and approved by UTHealth Animal Welfare Committee (AWC-16-0060 and AWC-19-0029).

## Author contributions

CW and HKQ conducted experiments, acquired data, analyzed data, and wrote the manuscript. TCJM, PS, WB, SDC, NW, JK, TW, EJW, NDE, and RPN conducted experiments, acquired data, and analyzed data. RPN and PJW analyzed data and provided research material. PM provided reagents. SSKJ, RAT, DR, and HJH provided research material and helped interpret clinical data. BFD and HLF analyzed and interpreted data and provided reagents.

## Supplementary Material

Supplemental data

## Version 1 04/14/2022

In-Press Preview

## Version 2 05/23/2022

Electronic publication

## Version 3 04/04/2023

The color legend was corrected for Figure 1A, 1B, and 1C

## References

[1] Lederer DJ, Martinez FJ (2018). Idiopathic pulmonary fibrosis. N Engl J Med.

[2] Richeldi L (2017). Idiopathic pulmonary fibrosis. Lancet.

[3] Hutchinson J (2015). Global incidence and mortality of idiopathic pulmonary fibrosis: a systematic review. Eur Respir J.

[4] Sharif R (2017). Overview of idiopathic pulmonary fibrosis (IPF) and evidence-based guidelines. Am J Manag Care.

[5] Raghu G (2011). An official ATS/ERS/JRS/ALAT statement: idiopathic pulmonary fibrosis: evidence-based guidelines for diagnosis and management. Am J Respir Crit Care Med.

[6] Baumgartner KB (2000). Occupational and environmental risk factors for idiopathic pulmonary fibrosis: a multicenter case-control study. Collaborating Centers. Am J Epidemiol.

[7] Dickey BF, Whitsett JA (2017). Understanding interstitial lung disease: it’s in the mucus. Am J Respir Cell Mol Biol.

[8] Seibold MA (2011). A common MUC5B promoter polymorphism and pulmonary fibrosis. N Engl J Med.

[9] Armanios MY (2007). Telomerase mutations in families with idiopathic pulmonary fibrosis. N Engl J Med.

[10] Konigshoff M (2008). Functional Wnt signaling is increased in idiopathic pulmonary fibrosis. PLoS One.

[11] Bolanos AL (2012). Role of Sonic Hedgehog in idiopathic pulmonary fibrosis. Am J Physiol Lung Cell Mol Physiol.

[12] Flozak AS (2010). Beta-catenin/T-cell factor signaling is activated during lung injury and promotes the survival and migration of alveolar epithelial cells. J Biol Chem.

[13] El-Hashash AH (2011). Six1 transcription factor is critical for coordination of epithelial, mesenchymal and vascular morphogenesis in the mammalian lung. Dev Biol.

[14] Kumar JP (2009). The sine oculis homeobox (SIX) family of transcription factors as regulators of development and disease. Cell Mol Life Sci.

[15] Blevins MA (2015). The SIX1-EYA transcriptional complex as a therapeutic target in cancer. Expert Opin Ther Targets.

[16] Liu Q (2016). The expression profile and clinic significance of the SIX family in non-small cell lung cancer. J Hematol Oncol.

[17] Xia Y (2014). miR-204 functions as a tumor suppressor by regulating SIX1 in NSCLC. FEBS Lett.

[18] Bauer Y (2015). A novel genomic signature with translational significance for human idiopathic pulmonary fibrosis. Am J Respir Cell Mol Biol.

[19] Sivakumar P (2019). RNA sequencing of transplant-stage idiopathic pulmonary fibrosis lung reveals unique pathway regulation. ERJ Open Res.

[20] Xu Y (2016). Single-cell RNA sequencing identifies diverse roles of epithelial cells in idiopathic pulmonary fibrosis. JCI Insight.

[21] Jenkins RG (2017). An Official American Thoracic Society Workshop Report: use of animal models for the preclinical assessment of potential therapies for pulmonary fibrosis. Am J Respir Cell Mol Biol.

[22] Headley L (2018). Low-dose administration of bleomycin leads to early alterations in lung mechanics. Exp Physiol.

[23] Darwiche T (2019). Alterations in cardiovascular function in an experimental model of lung fibrosis and pulmonary hypertension. Exp Physiol.

[24] Williamson JD (2015). The pathogenesis of bleomycin-induced lung injury in animals and its applicability to human idiopathic pulmonary fibrosis. Exp Lung Res.

[25] Naikawadi RP (2016). Telomere dysfunction in alveolar epithelial cells causes lung remodeling and fibrosis. JCI Insight.

[26] Lu K (2013). Abrogation of Eya1/Six1 disrupts the saccular phase of lung morphogenesis and causes remodeling. Dev Biol.

[27] Gui YS (2012). SPC-Cre-ERT2 transgenic mouse for temporal gene deletion in alveolar epithelial cells. PLoS One.

[28] Le Grand F (2012). Six1 regulates stem cell repair potential and self-renewal during skeletal muscle regeneration. J Cell Biol.

[29] Trawinska MA (2016). Patient considerations and drug selection in the treatment of idiopathic pulmonary fibrosis. Ther Clin Risk Manag.

[30] McCoy EL (2009). Six1 expands the mouse mammary epithelial stem/progenitor cell pool and induces mammary tumors that undergo epithelial-mesenchymal transition. J Clin Invest.

[31] Coletta RD (2008). Six1 overexpression in mammary cells induces genomic instability and is sufficient for malignant transformation. Cancer Res.

[32] Lee JM (2018). Involvement of alveolar epithelial cell necroptosis in idiopathic pulmonary fibrosis pathogenesis. Am J Respir Cell Mol Biol.

[33] Ahmed M (2012). Eya1-Six1 interaction is sufficient to induce hair cell fate in the cochlea by activating Atoh1 expression in cooperation with Sox2. Dev Cell.

[34] Chai L (2006). Transcriptional activation of the SALL1 by the human SIX1 homeodomain during kidney development. J Biol Chem.

[35] Olivieri C (2016). Macrophage migration inhibitory factor in lung tissue of idiopathic pulmonary fibrosis patients. Exp Lung Res.

[36] Bargagli E (2009). Analysis of macrophage migration inhibitory factor (MIF) in patients with idiopathic pulmonary fibrosis. Respir Physiol Neurobiol.

[37] Xue YM (2018). Macrophage migration inhibitory factor promotes cardiac fibroblast proliferation through the Src kinase signaling pathway. Mol Med Rep.

[38] Ningyan G (2015). The role of macrophage migration inhibitory factor in mast cell-stimulated fibroblast proliferation and collagen production. PLoS One.

[39] Lan H (2020). MIF signaling blocking alleviates airway inflammation and airway epithelial barrier disruption in a HDM-induced asthma model. Cell Immunol.

[40] Selman M (2008). Idiopathic pulmonary fibrosis: aberrant recapitulation of developmental programs?. PLoS Med.

[41] Kugler MC (2015). Sonic hedgehog signaling in the lung. From development to disease. Am J Respir Cell Mol Biol.

[42] Iwanaga R (2012). Expression of Six1 in luminal breast cancers predicts poor prognosis and promotes increases in tumor initiating cells by activation of extracellular signal-regulated kinase and transforming growth factor-beta signaling pathways. Breast Cancer Res.

[43] Jin H (2014). Sineoculis homeobox homolog 1 protein is associated with breast cancer progression and survival outcome. Exp Mol Pathol.

[44] Yang ZC (2018). MiR-448-5p inhibits TGF-β1-induced epithelial-mesenchymal transition and pulmonary fibrosis by targeting Six1 in asthma. J Cell Physiol.

[45] Yang ZC (2016). Targeted inhibition of Six1 attenuates allergic airway inflammation and remodeling in asthmatic mice. Biomed Pharmacother.

[46] Adams TS (2020). Single-cell RNA-seq reveals ectopic and aberrant lung-resident cell populations in idiopathic pulmonary fibrosis. Sci Adv.

[47] Reyfman PA (2019). Single-cell transcriptomic analysis of human lung provides insights into the pathobiology of pulmonary fibrosis. Am J Respir Crit Care Med.

[48] Habermann AC (2020). Single-cell RNA sequencing reveals profibrotic roles of distinct epithelial and mesenchymal lineages in pulmonary fibrosis. Sci Adv.

[49] McDonough JE (2019). Transcriptional regulatory model of fibrosis progression in the human lung. JCI Insight.

[50] Morse C (2019). Proliferating SPP1/MERTK-expressing macrophages in idiopathic pulmonary fibrosis. Eur Respir J.

[51] Marsh LM (2009). Surface expression of CD74 by type II alveolar epithelial cells: a potential mechanism for macrophage migration inhibitory factor-induced epithelial repair. Am J Physiol Lung Cell Mol Physiol.

[52] Bernhagen J (2007). MIF is a noncognate ligand of CXC chemokine receptors in inflammatory and atherogenic cell recruitment. Nat Med.

[53] Gunther S (2019). Role of MIF and D-DT in immune-inflammatory, autoimmune, and chronic respiratory diseases: from pathogenic factors to therapeutic targets. Drug Discov Today.

[54] Miller EJ (2008). Macrophage migration inhibitory factor stimulates AMP-activated protein kinase in the ischaemic heart. Nature.

[55] Günther S (2018). Macrophage migration inhibitory factor (MIF) inhibition in a murine model of bleomycin-induced pulmonary fibrosis. Int J Mol Sci.

[56] Tanino Y (2002). Role of macrophage migration inhibitory factor in bleomycin-induced lung injury and fibrosis in mice. Am J Physiol Lung Cell Mol Physiol.

[57] Das R (2011). Role of macrophage migration inhibitory factor in the Th2 immune response to epicutaneous sensitization. J Clin Immunol.

[58] Chen PF (2010). ISO-1, a macrophage migration inhibitory factor antagonist, inhibits airway remodeling in a murine model of chronic asthma. Mol Med.

[59] Amano T (2007). Blockade of macrophage migration inhibitory factor (MIF) prevents the antigen-induced response in a murine model of allergic airway inflammation. Inflamm Res.

[60] Kobayashi M (2006). Role of macrophage migration inhibitory factor in ovalbumin-induced airway inflammation in rats. Eur Respir J.

[61] Mizue Y (2005). Role for macrophage migration inhibitory factor in asthma. Proc Natl Acad Sci U S A.

[62] Kolb P (2020). The importance of interventional timing in the bleomycin model of pulmonary fibrosis. Eur Respir J.

[63] Moeller A (2008). The bleomycin animal model: a useful tool to investigate treatment options for idiopathic pulmonary fibrosis?. Int J Biochem Cell Biol.

[64] Patrick AN (2013). Structure-function analyses of the human SIX1-EYA2 complex reveal insights into metastasis and BOR syndrome. Nat Struct Mol Biol.

[65] Zhao Y (2015). Small-molecule inhibitors of the MDM2-p53 protein-protein interaction (MDM2 Inhibitors) in clinical trials for cancer treatment. J Med Chem.

[66] Vogler M (2009). Bcl-2 inhibitors: small molecules with a big impact on cancer therapy. Cell Death Differ.

[67] Daum N (2012). Isolation, cultivation, and application of human alveolar epithelial cells. Methods Mol Biol.

[68] Corti M (1996). Isolation and primary culture of murine alveolar type II cells. Am J Respir Cell Mol Biol.

[69] Manika K (2012). The impact of pulmonary arterial pressure on exercise capacity in mild-to-moderate cystic fibrosis: a case control study. Pulm Med.

[70] Wikenheiser KA (1993). Production of immortalized distal respiratory epithelial cell lines from surfactant protein C/simian virus 40 large tumor antigen transgenic mice. Proc Natl Acad Sci U S A.

[71] Collum SD (2017). Inhibition of hyaluronan synthesis attenuates pulmonary hypertension associated with lung fibrosis. Br J Pharmacol.

[72] Thery C (2018). Minimal information for studies of extracellular vesicles 2018 (MISEV2018): a position statement of the International Society for Extracellular Vesicles and update of the MISEV2014 guidelines. J Extracell Vesicles.

