# Supplementary material for: Sine oculis homeobox homolog 1 plays a critical role in pulmonary fibrosis
Source: JCI Insight. 2022 May 23;7(10):e142984. doi: 10.1172/jci.insight.142984 (PMC9220956; doi:10.1172/jci.insight.142984)

Supplemental Materials

Supplemental Figure 1. Mice overexpressing SIX1 in AT2 cells have worse lung function following BLM treatment. Lung function parameters for single frequency elastase (Ers, **A**), single frequency resistance (Rrs, **B**), tissue damping (G, **C**), tissue elastance (H, **D**), quasi-static compliance (Cst, **E**), inspiratory capacity (IC, **F**), Forced Expiratory volume in 0.1 s (FEV ,**G**), and Forced Vital Capacity (FVC, **H**) from anesthetized BLM treated *SPC*-rtTA (N=19) or *SIX*TET mice (N=6) mice. Significance levels * $P \leq 0.05$ and ** $P \leq 0.01$; refer to Student's t-test with Welch correction.

Supplemental Figure 2. SIX1 depletion in MLE12 cells increases proliferation *in vitro*. A) RT-qPCR of MLE12 cells overexpressing SIX1 (*Six1*OE), and *Six1*-siRNA (siRNA) compared to GFP-expressing (GFP) controls. **B**) WST-1 assay using MLE12 cells overexpressing SIX1 (*Six1*OE), and *Six1*-siRNA (siRNA) compared to GFP-expressing (GFP)controls. **C**) Representative images (scale bars in white, 100µm) of pHH3 staining (green signal) in MLE12 cells with either SIX1 overexpression (*Six1*) and *Six1* siRNA (siRNA) treated cells for 48 hrs with quantification of phosphorylated histone H3 at serine 10 (pHH3). All samples were counterstained with DAPI (blue signal). **D**) Comparing positive pHH3 signal per DAPI signal using an automated fluorescence cell cytometer. Significance levels * $P \leq 0.05$ and ** $P \leq 0.01$; and *** $P \leq 0.001$, refer Student's t-tests with Welch correction. N=3 for all groups in Panels A and D. N=19 (GFP), N=28 (*Six1*OE) and N=16 for si*Six1* for Panel B.

Supplemental Figure 3. Volcano Plot for SIX1 OE RNA-seq

Volcano plot showing False Discovery Rate (FDR, Log10[P-value]) vs Fold Change for genes associated with an FDR over 2 was considered significant. Mif and Six1 are labelled and plotted in teal.

Supplemental Figure 4. RNAscope signals for SIX1 in non-SPC positive cells

Dual RNAscope for SIX1 (teal) and SPC (magenta) from 5 IPF patients. Black arrows denote signals for SIX1 in airway epithelial and mesenchymal cells. Scale bar represents 100 μm.

Supplemental Table 1. Human lung donor information. Data is shown as mean ± SEM. BMI, body mass index; FEV1, forced expiration volume over 1 second; FVC, forced vital capacity; IPF, idiopathic pulmonary fibrosis; COPD, chronic obstructive pulmonary disease. Control lungs were collected from lung explants rejected for lung transplantation with a PaO2:FiO2 ratio > 300. Data is shown as mean ± SEM. BMI, body mass index; FEV1, forced expiration volume over 1 second; FVC, forced vital capacity; IPF, idiopathic pulmonary fibrosis; COPD, chronic obstructive pulmonary disease.

	**CONTROL**	**COPD**	**IPF**
**AGE (years)**	42 ± 20.4	64.4 ± 6.0	66.4 ± 1.9
**GENDER (female/male)**	4/7	10/8	7/14
**HEIGHT (cm)**	164.4 ± 37.2	170.6 ± 10.6	170.8 ± 10.7
**WEIGHT (Kg)**	78.2 ± 20.3	74.2 ± 16.2	83.4 ± 16.0
**BMI**	26.5 ± 6.2	24.5 ± 5.5	29.0 ± 6.0
**FVC (%)**	-	52.4 ± 11.5	48.6 ± 17.6
**FEV1 (%)**	-	23.9 ± 8.4	51.7 ± 19.5

Supplemental Table 2. Primary and Secondary Antibodies

Primary and secondary antibodies used for both mouse and human experiments with manufacturer, target, species, catalog numbers, and dilutions used for either immunoblot (WB) or immunohistochemistry/immunofluorescence (IHC/IF).

Antibody	Source	Target	Provider	Catalog	Dilution
MIF	mouse	mouse	abcam	ab65869	1:1000 (WB)
Col1a1	Rabbit	hu, nim rat	Proteintech	25870-1-AP	1:1000 (WB)
aSMA	rabbit	hu,mo,rat	abcam	ab5694	1:1000 (IHC)
SIX1	rabbit	hu,mo	Novus biologicals	NBP1-84264	1:100 (IHC/IF)
SIX1	rabbit	hu,mo,rat	CST	12891S	1:100 (IHC/IF) 1:1000 (WB)
ß-actin	rabbit	hu,mo,rat	CST	4967S	1:3000 (WB)
SIX1	rabbit	hu,mo	Sigma Aldrich	HPA001893	1:100 (IF)
EYA1	mouse	hu	Abnova	H00002138-A01	1:1000 (WB)
EYA2	rabbit	hu	Abcam	ab92505	1:3000 (WB)

SPC	rabbit	hu,mo,rat	Millipore	Ab3786	1:200 (IHC) 1:1000 (IF)
EYA2	rabbit	hu,mo	Thermo Scientific	PA5-68561	1:10,000 (WB)
Alexa Fluor 488	rabbit	mouse	Thermo Scientific	A11059	1:1000 (IF)
Alexa Fluor 594	goat	rabbit	Thermo Scientific	A11012	1:1000 (IF)
Alexa Fluor 488	goat	rabbit	Thermo Scientific	A11008	1:1000 (IF)
Alexa Fluor 633	goat	mouse	Thermo Scientific	A21052	1:1000 (IF)
Alexa Fluor 594	donkey	goat	Thermo Scientific	R37119	1:500 (IF)
Alexa Fluor 488	donkey	rabbit	Thermo Scientific	R37114	1:500 (IF)

Supplemental Table 3. Human and Mouse Primers

Human and Mouse primers used with gene name and forward (FW/F1) and reverse (RV/R1) sequences.

**Gene**	**Primer name**	**Sequence**
GAPDH	GAPDH_homo_FW	AAGGTGAAGGTCGGAGTCAAC
GAPDH	GAPDH_homo_RV	GGGGTCATTGATGGCAACAATA
18S	18S_homo_mus_FW	GTAACCCGTTGAACCCCATT
18S	18S_homo_mus_RV	CCATCCAATCGGTAGTAGCG
EYA2	EYA2_homo_FW	CTACCAGATGCACGGCACAA
EYA2	EYA2_homo_RV	AGCCGGGGTAGGAAGGATAG
EYA4	EYA4_homo_FW	TCTGATTCTGTGCACGTTTTCT
EYA4	EYA4_homo_RV	CTACTTGGGAGTGGCAGGAG
EYA1	EYA1_homo_FW	TCCATGACTCCCAATGGCAC
EYA1	EYA1_homo_RV	GGGTATGGTCTGTTGGAAGGG
FN1	FN1_homo_FW	GGTGGAATAGAGCTCCCAGG
FN1	FN1_homo_RV	GCAGCCTGCATCTGAGTACA
SIX1	SIX1_homo_FW	CTGCCGTCGTTTGGCTTTAC
SIX1	SIX1_homo_RV	GCTCTCGTTCTTGTGCAGGT
SIX2	SIX2_homo_FW	CGGGTTGTGGCTGTTAGAAT
SIX2	SIX2_homo_RV	CACCACACAGGTCAGCAACT
SIX4	SIX4_homo_FW	GGGAGCAAGAGAGCTCAAGA
SIX4	SIX4_homo_RV	GTCAGTGGCAGCTTCACAAG
SIX6	SIX6_homo_FW	CTGTGACAGGACCTGCTGC

SIX6	SIX6_homo_RV	CAACCGGACTGACCCCTAC
SP-C	SP-C_homo_FW	CGATAAGAAGGCGTTTCAGG
SP-C	SP-C_homo_RV	AGCAAAGAGGTCCTGATGGA
Col1A1	Col1A1_mus_F2	GGTTTCCACGTCTCACCATT
Col1A1	Col1A1_mus_R2	CGGCTCCTGCTCCTCTTAG
Col1A2	Col1A2_mus_F1	AGCAGGTCCTTGGAAACCTT
Col1A2	Col1A2_mus_R1	AAGGAGTTTCATCTGGCCCT
Six1	Six1_mus_FW	GAAAGGGAGAACACCGAAAACA
Six1	Six1_mus_RV	GTGGCCCATATTGCTCTGGA
MIF	MIF_homo_FW	GAACAACTCCACCTTCGCCT
MIF	MIF_homo_RV	CCGTTTATTTCTCCCCACCA
Dach1	Dach1_mus_F1	CTTAGGAGGCCTTCCAGGTC
Dach1	Dach1_mus_R1	GAACCGCTGCAAACTCATCT
18S	18S_homo_mus_FW	GTAACCCGTTGAACCCCATT
18S	18S_homo_mus_RV	CCATCCAATCGGTAGTAGCG
Eya2	Eya2_mus_F1	GATAATCCTGGTGCACGCTC
Eya2	Eya2_mus_R1	CAGAGCCCCTACACCTACCC
Eya4	Eya2_mus_F3	AACCCAGCTGATTCCTGCTC
Eya4	Eya2_mus_R3	GGCATGTTGTGCTGGTTAGC
Eya3	Eya3_mus_F1	GGACTGAATTGCAGGTCTCTG
Eya3	Eya3_mus_R1	GTTCCAGAGTGGGTCCGTAA
Fn1	Fn1_mus_F1	ACTGGATGGGGTGGGAAT
Fn1	Fn1_mus_R1	GGAGTGGCACTGTCAACCTC

CD74	CD74_ Mus_F1	AGTGCGACGAGAACGGTAAC
CD74	CD74_ Mus_R1	CGTTGGGGAACACACACCA
Six4	Six4_mus_F2	CTGTGGCTGGCTCACTTGTA
Six4	Six4_mus_R2	GGAGCATTGGATTCTCTCCA
Sftpc	Sftpc_mus_F1	ATGAGAAGGCGTTTGAGGTG
Sftpc	Sftpc_mus_R1	AGCAAAGAGGTCCTGATGGA
CXCR4	CXCR4_ Mus_F1	GAAGTGGGGTCTGGAGACTAT
CXCR4	CXCR4_ Mus_R1	TTGCCGACTATGCCAGTCAAG
MIF	MIF 1_ Mus_F1	GCCAGAGGGGTTTCTGTCG
MIF	MIF 1_ Mus_R1	GTTCGTGCCGCTAAAAGTCA
MMP11	MMP11_ Mus_F1	CCGGAGAGTCACCGTCATC
MMP11	MMP11_ Mus_R1	GCAGGACTAGGGACCCAATG

Supplementary Figure 1

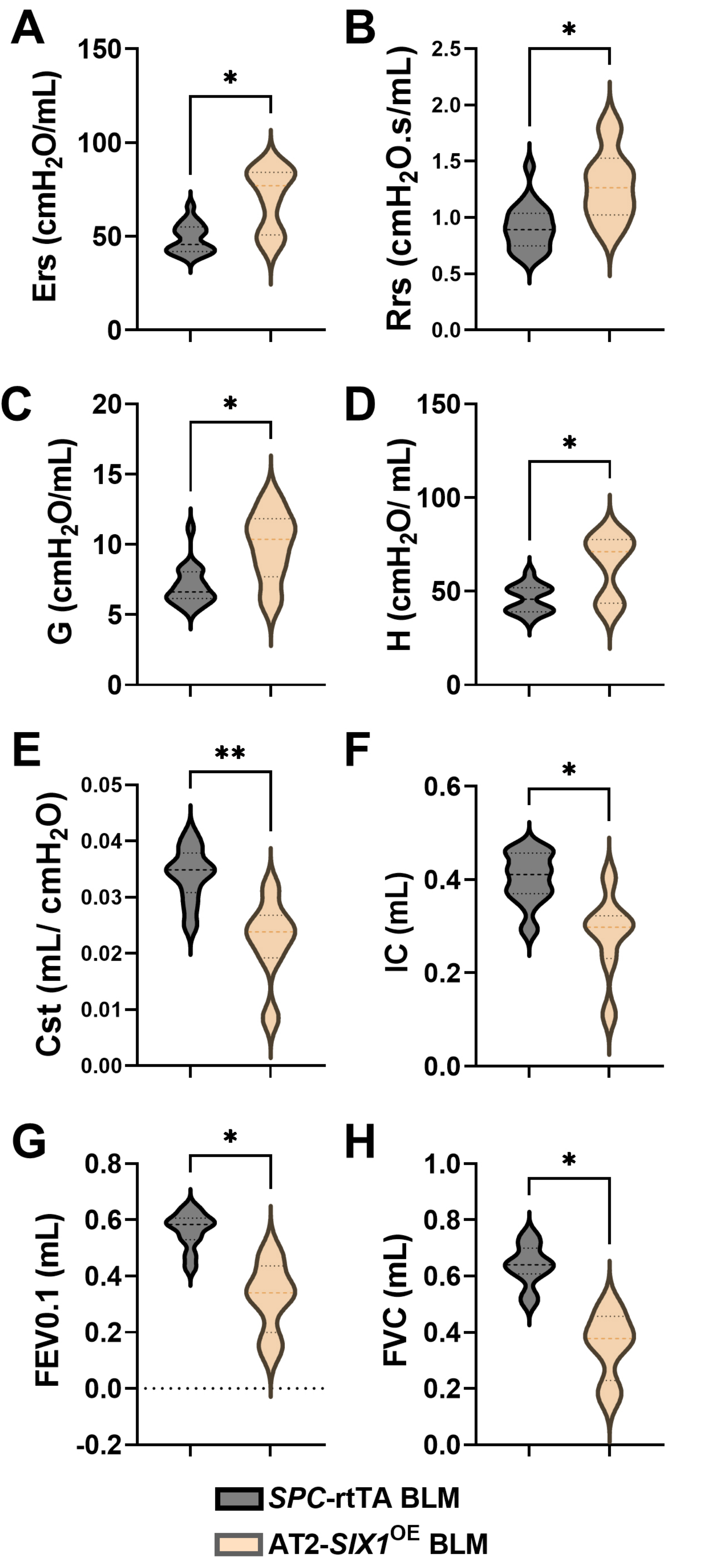

Supplementary Figure 2

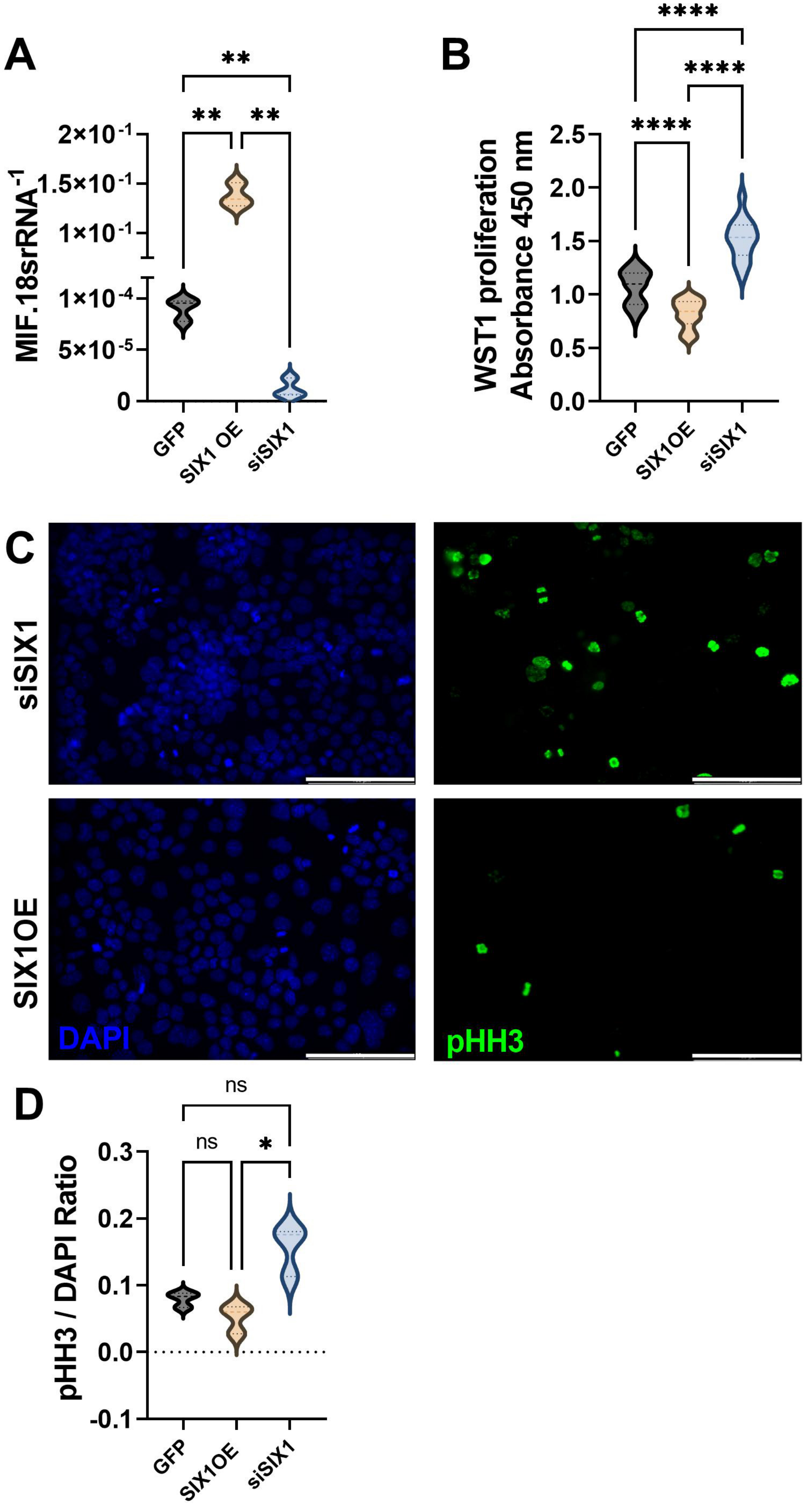

Supplementary Figure 3

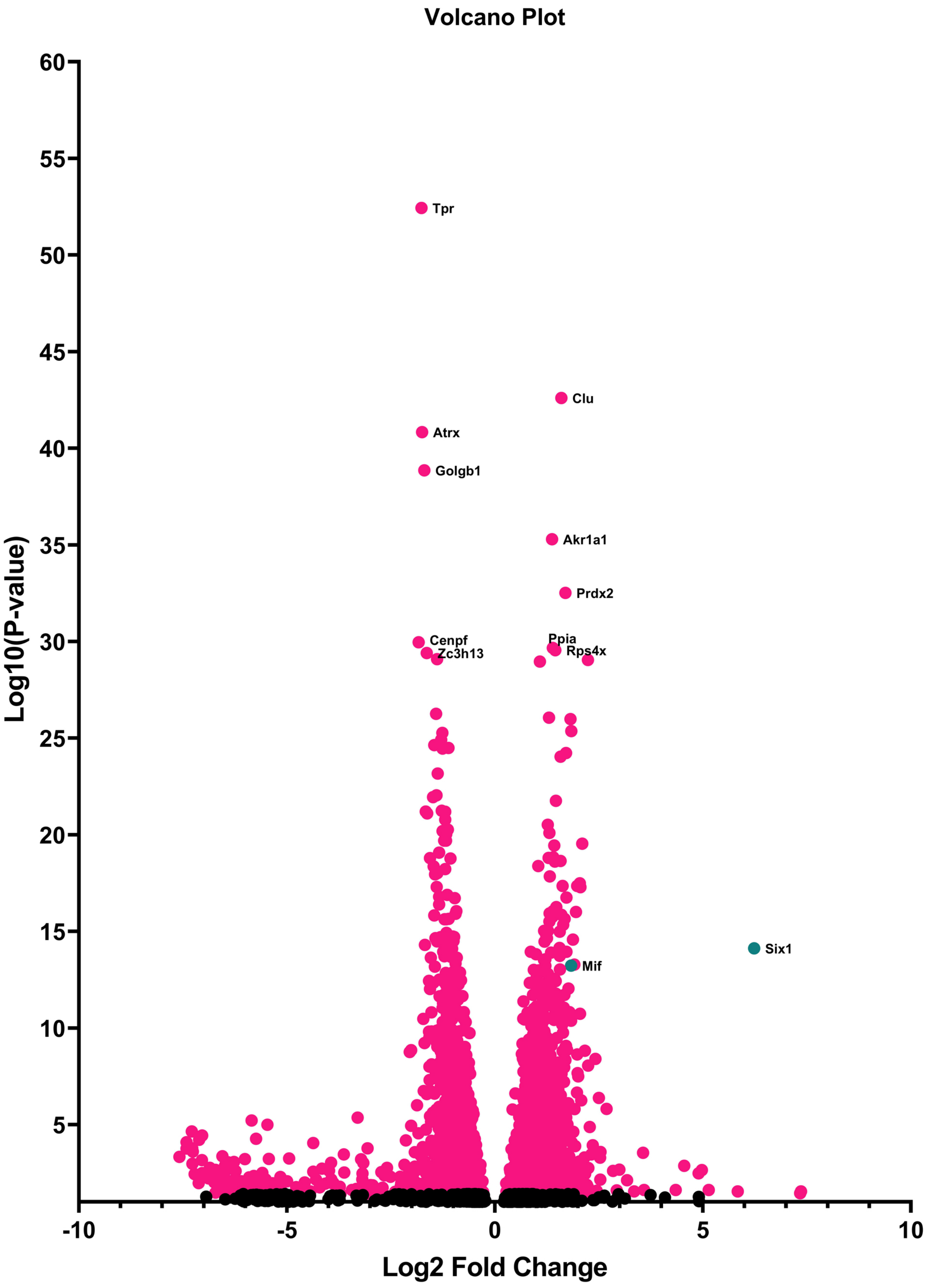

Supplementary Figure 4

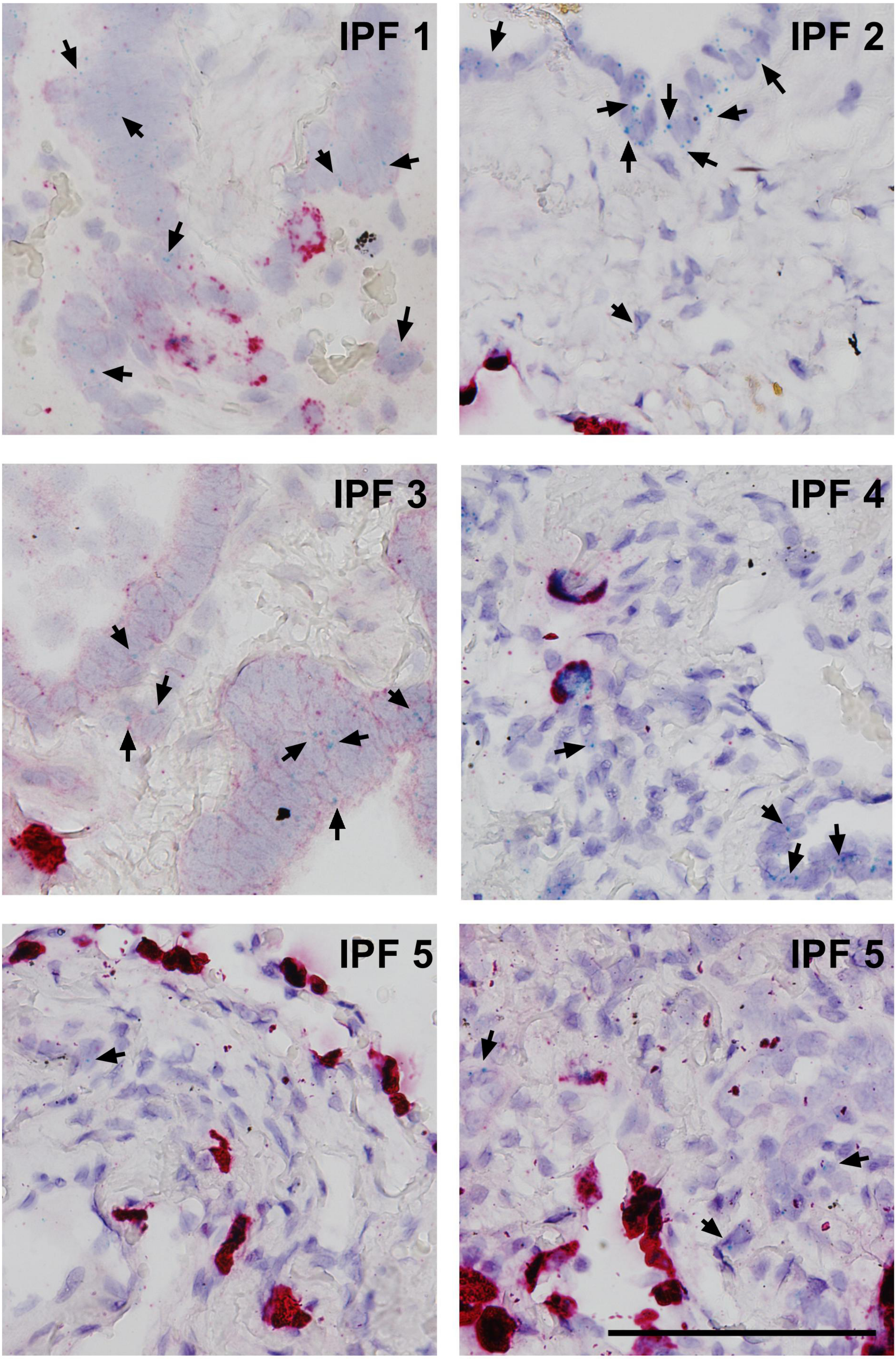

Supplement: Supplemental data [file jciinsight-7-142984-s012.pdf]
